# Development and validation of prediction models to estimate risk of primary total hip and knee replacements using data from the UK: two prospective open cohorts using the UK Clinical Practice Research Datalink

**DOI:** 10.1136/annrheumdis-2018-213894

**Published:** 2018-10-18

**Authors:** Dahai Yu, Kelvin P Jordan, Kym I E Snell, Richard D Riley, John Bedson, John James Edwards, Christian D Mallen, Valerie Tan, Vincent Ukachukwu, Daniel Prieto-Alhambra, Christine Walker, George Peat

**Affiliations:** 1 Arthritis Research UK Primary Care Centre, Research Institute for Primary Care & Health Sciences, Keele University, Keele, UK; 2 Centre for Prognostic Research, Arthritis Research UK Primary Care Centre, Research Institute for Primary Care & Health Sciences, Keele University, Keele, UK; 3 GREMPAL (Grup de Recerca en Epidemiologia de les Malalties Prevalents de l’Aparell Locomotor), Idiap Jordi Gol Primary Care Research Institute and CIBERFes, Universitat Autònoma de Barcelona and Instituto de Salud Carlos III, Barcelona, Spain; 4 Musculoskeletal Pharmaco- and Device Epidemiology – Centre for Statistics in Medicine, Nuffield Department of Orthopaedics, Rheumatology, and Musculoskeletal Sciences, University of Oxford, Oxford, UK

**Keywords:** osteoarthritis, total hip replacement, total knee replacement, primary care, electronic health record, record-wide association study, risk prediction model

## Abstract

**Objectives:**

The ability to efficiently and accurately predict future risk of primary total hip and knee replacement (THR/TKR) in earlier stages of osteoarthritis (OA) has potentially important applications. We aimed to develop and validate two models to estimate an individual’s risk of primary THR and TKR in patients newly presenting to primary care.

**Methods:**

We identified two cohorts of patients aged ≥40 years newly consulting hip pain/OA and knee pain/OA in the Clinical Practice Research Datalink. Candidate predictors were identified by systematic review, novel hypothesis-free ‘Record-Wide Association Study’ with replication, and panel consensus. Cox proportional hazards models accounting for competing risk of death were applied to derive risk algorithms for THR and TKR. Internal–external cross-validation (IECV) was then applied over geographical regions to validate two models.

**Results:**

45 predictors for THR and 53 for TKR were identified, reviewed and selected by the panel. 301 052 and 416 030 patients newly consulting between 1992 and 2015 were identified in the hip and knee cohorts, respectively (median follow-up 6 years). The resultant model C-statistics is 0.73 (0.72, 0.73) and 0.79 (0.78, 0.79) for THR (with 20 predictors) and TKR model (with 24 predictors), respectively. The IECV C-statistics ranged between 0.70–0.74 (THR model) and 0.76–0.82 (TKR model); the IECV calibration slope ranged between 0.93–1.07 (THR model) and 0.92–1.12 (TKR model).

**Conclusions:**

Two prediction models with good discrimination and calibration that estimate individuals’ risk of THR and TKR have been developed and validated in large-scale, nationally representative data, and are readily automated in electronic patient records.

**Key messages**

**What is already known about this subject?**

- The majority of primary total hip replacement (THR) and primary total knee replacement (TKR) were performed for patients with end-stage osteoarthritis in the UK, but clinical risk predictions of THR and TKR in patients who newly presenting hip pain/osteoarthritis and knee pain/osteoarthritis at the primary care settings have not been developed.

**What does this study add?**

- A novel approach for predictor selection has been developed. Two risk prediction models based on clinical variables that are available in the UK primary care electronic health record have been developed and validated. These algorithms can be used to inform clinical decision making, for example targeting intensive non-surgical management at patients identified at high risk of future primary THR or TKR.

**How might this impact on clinical practice?**

- These algorithms can be used to inform clinical decision making, for example targeting intensive non-surgical management at patients identified at high risk of future primary THR or TKR.

## Introduction

Osteoarthritis (OA) is the most common form of arthritis and a leading cause of disability in populations worldwide.^1^ Although characterised as a slowly progressive condition, recent studies have highlighted substantial heterogeneity between groups of patients in the course of symptoms,^2–4^ function^5^ and structural disease.^6^ Healthcare costs attributed to OA, driven largely by primary and revision arthroplasty,^7^ appear concentrated in a minority of patients.^8^ The development and application of prognostic models capable of identifying patients with OA at high risk of future progression is now recognised as a priority internationally and by patients, carers and health and social care professionals.^9^ Such models could have important clinical and research applications: better targeting of intensive non-surgical care; selection of patients for active monitoring; timely assessment and discussion of appropriateness for referral and recruitment of ‘high-risk’ patients as part of efficient clinical trial design evaluating new secondary prevention treatments.

Models that rely on pooling data from existing clinical trials and bespoke cohorts may offer the prospect of carefully measured, highly relevant predictors and outcomes, but are limited by the availability of large, long-term studies with sufficient harmonised data. Furthermore, due to the high prevalence of OA and to time and cost constraints, models that require the collection of biomarkers, imaging or lengthy patient-reported instruments are unlikely to be implemented at scale in routine primary care, irrespective of their informativeness in research settings. An alternative approach, and the one chosen in our study, is to investigate whether data already routinely available in large, representative primary electronic healthcare databases could provide accurate predictions which are feasible for implementing in routine primary care. This approach has been used to derive and validate risk algorithms for condition-specific outcomes in other chronic non-communicable diseases and for complex events such as hospital admissions.^10–18^

We sought to develop and validate multivariable prediction models, based exclusively on information routinely recorded within the primary care electronic health record, to estimate the risk of primary total hip replacement (THR) and total knee replacement (TKR) in patients newly presenting with hip pain/OA and knee pain/OA in UK primary care. To achieve this, we included a novel approach to identify candidate prognostic factors recorded in the primary care patient record.

## Methods

### Data source and study population

We used data from the Clinical Practice Research Datalink (CPRD) covering a representative sample of 7% of the UK general population.^19^ The definition^20^ and the selection of population were presented in online supplementary technical appendix, figures S1 and S2.

10.1136/annrheumdis-2018-213894.supp1 Supplementary data

### Defining THR/TKR

Primary THR and TKR were identified within CPRD using the Read code list developed and applied in CPRD by Culliford and colleagues^21^ and validated by Hawley *et al*.^22 23^ Details of outcome definition was presented in online supplementary technical appendix.

### Candidate predictors

Candidate predictors were identified from three sources: (i) a systematic review of previously published studies (further details available in online supplementary technical appendix); (ii) potentially relevant general predictors used within 12 QResearch risk algorithms and shown to be feasibly obtained from UK primary care (eg, sociodemographic, lifestyle related, comorbidities)^10–18 24–26^ (iii) a hypothesis-free record-wide association study (ReWAS) of all third-level Read morbidity and process of care codes and for prescribed medicine, third-level sections within the British National Formulary which had been recorded in ≥1% of cases in the 3 years prior to date of arthroplasty. There were 6109 third-level Read morbidity and process of care codes and 325 prescribed medications assessed. The ReWAS case-control analysis was conducted in CPRD, with replication of ‘hits’ in a separate UK regional primary care Electronic Health Record (EHR) dataset—Consultations in Primary Care Archive (further details available in online supplementary technical appendix). Morbidities, processes of care and prescribed medications that were statistically significantly associated with THR or TKR in the screen were taken forward (online supplementary figures S3 and S5 for TKR; online supplementary figures S4 and S6 for THR). The candidate predictors were assessed for clinical relevance by a review panel including seven members (six clinicians and one lay member), and the predictors agreed as relevant by ≥4 members were included in the modelling stage (online supplementary table S1). This process identified 29 candidate predictors for THR and 34 for TKR. These were extracted from records in the 3 years prior to index consultation.

### Statistical analysis for model derivation

Primary THR and TKR occurring since the patients’ index consultation for hip pain/OA and knee pain/OA in primary care were treated as time-to-event outcomes in the THR and TKR models, respectively. Statistical method of predictor selection was presented in online supplementary technical appendix.

We formed the risk (cumulative incidence) equations for predicting an individual’s 10-year probability of primary THR and TKR since the index consultation for hip pain/OA and knee pain/OA, by using the developed model’s baseline cumulative incidence function (CIF) at 10 years, along with the estimated regression coefficients (β) and the individual’s predictor values (**X**) using the following equation^27^:

$$\hat{CIF}\left(t=10\right)=1-{\left(1-{\hat{CIF}}_{0}(t=10)\right)}^{exp(X\hat{\beta })}$$

### Validation of prediction models

We assessed the model discrimination using Harrell’s C-statistic and the model calibration using calibration slope (details in online supplementary technical appendix)^28–30^ over the 10 years of follow-up.

We assessed the apparent performance of the models; that is, the observed performance in exactly the same data used to develop the model. However, we also used an internal–external cross-validation (IECV) approach (online supplementary technical appendix) to evaluate the two derived prediction models over 13 geographical regions in the UK (presented in table 1).^31–33^

**Table 1:** Characteristics of study populations for the primary total knee replacement (TKR) model and primary total hip replacement (THR) models

Predictor	THR model	TKR model
	N=301 052	N=416 030
Outcome, n (%)	15 509 (5.15)	18 289 (4.40)
Median follow-up duration (range), years	6.27 (2.00 to 24.49)	6.21 (2.00 to 24.56)
Gender (female)	191 288 (63.54)	238 549 (57.34)
Ethnicity		
White	92 269 (30.65)	125 982 (30.28)
Other ethnicity group	3621 (1.20)	6001 (1.44)
Not recorded	205 162 (68.15)	284 047 (68.28)
Region		
North East	6552 (2.18)	8647 (2.08)
North West	38 618 (12.83)	51 331 (12.34)
Yorkshire and the Humber	13 140 (4.36)	17 282 (4.15)
East Midlands	12 779 (4.24)	16 670 (4.00)
West Midlands	29 273 (9.72)	40 383 (9.71)
East of England	26 194 (8.70)	36 488 (8.77)
South West	24 885 (8.27)	34 216 (8.22)
South Central	32 048 (10.65)	46 736 (11.32)
London	23 541 (7.82)	35 197 (8.46)
South East Coast	28 760 (9.55)	41 608 (10.00)
Northern Ireland	11 445 (3.80)	14 708 (3.54)
Scotland	25 672 (8.53)	34 547 (8.30)
Wales	28 145 (9.35)	38 217 (9.18)
Family history of arthritis/ osteoarthritis (OA)	1862 (0.62)	2135 (0.51)
Smoking status		
Light smoker	6845 (2.27)	8502 (2.04)
Moderate/heavy smoker	34 896 (11.59)	48 398 (11.63)
Drinking status		
Ex-drinker	11 069 (3.68)	13 924 (3.35)
Light drinker	212 058 (70.44)	294 273 (70.73)
Moderate drinker	4172 (1.39)	6523 (1.57)
Heavy drinker	1945 (0.65)	3177 (0.76)
Physical activity		
Taking light physical activity	26 855 (8.92)	44 876 (10.53)
Taking moderate physical activity	17 526 (5.82)	29 665 (7.13)
Taking heavy physical activity	1984 (0.66)	3229 (0.78)
Having diet consultation	110 780 (36.80)	173 969 (41.82)
Asthma	–	59 326 (14.26)
COPD	–	20 114 (4.83)
Chronic liver disease	6962 (2.31)	10 798 (2.60)
Diabetes mellitus	42 352 (14.07)	54 339 (13.06)
Malabsorption	1488 (0.49)	2091 (0.50)
Inflammatory bowel disease	16 863 (5.60)	26 722 (6.42)
Dementia	1265 (0.42)	2710 (0.65)
Cerebral palsy	153 (0.05)	205 (0.05)
Multiple sclerosis	942 (0.31)	1280 (0.31)
Cerebrovascular disease	11 819 (3.93)	16 150 (3.88)
Mental disorder		
Anxiety	38 133 (12.67)	56 163 (13.50)
Depression	48 558 (16.13)	36 034 (8.66)
Rheumatoid arthritis	5031 (1.67)	10 200 (2.45)
Systemic lupus erythematosus	673 (0.22)	865 (0.21)
Falls	29 780 (9.89)	49 859 (11.98)
Previous hip injury for THR model/previous knee injury for TKR model	7115 (2.36)	26 735 (6.43)
Osteoporosis	12 953 (4.30)	19 481 (4.68)
Knee effusion	–	6243 (1.50)
Diabetic foot	13 915 (4.62)	–
Bleed	44 812 (14.89)	70 543 (16.96)
Scoliosis/kyphosis	2340 (0.78)	3292 (0.79)
Development dysplasia of the hip	67 (0.02)	76 (0.02)
Chondrocalcinosis	781 (0.26)	1588 (0.38)
Recorded diagnosis of joint-specific OA		
Hip OA for TKR model/knee OA for THR model	272 (0.09)	26 640 (6.40)
Hand OA	9897 (3.29)	12 419 (2.99)
Generalised OA	7733 (2.57)	10 715 (2.58)
Other joint OA	12 380 (4.11)	151 282 (3.64)
Recorded diagnosis of non-specific OA	123 810 (41.13)	147 103 (35.36)
Low back pain	150 759 (50.08)	218 702 (52.57)
Hypertension	101 999 (33.88)	149 307 (35.89)
Atrial fibrillation	9842 (3.27)	16 515 (3.97)
Congestive cardiac failure	6397 (2.12)	9661 (2.32)
Venous thromboembolism	8040 (2.67)	12 245 (2.94)
Valvular heart disease	4157 (1.38)	6823 (1.64)
Joint injection	–	42 434 (10.20)
Knee arthroscopy	–	8310 (2.00)
ACL reconstruction	–	430 (0.10)
Phenytoin	819 (0.27)	1248 (0.30)
Physiotherapy	26 972 (8.96)	41 547 (9.99)
Corticosteroids	–	62 127 (14.93)
Glucocosteroids	32 203 (10.70)	51 645 (12.41)
Antidepressant	109 062 (36.23)	159 899 (38.43)
Analgesics		
Weak combination opioids	192 249 (63.86)	268 421 (64.52)
Moderate combination opioids	3231 (1.07)	4837 (1.16)
Strong/very strong combination opioids	3034 (1.01)	6359 (1.53)
Hormone treatment	82 221 (27.31)	107 321 (25.78)
Bisphosphonates	15 249 (5.07)	24 147 (5.80)
Topical NSAIDS		
NSAIDS	68 002 (22.59)	112 705 (27.09)
Other	3627 (1.20)	7422 (1.78)
Drugs for rheumatoid disease and gout		
NSAIDS	183 107 (60.82)	268 850 (64.62)
COX2	20 161 (6.70)	26 748 (6.43)
Prostaglandins and oxytocics	10 359 (3.44)	15 837 (3.80)
Rheumatoid factor test	876 (0.29)	1124 (0.27)
Age, mean±SD, years	62.98±12.17	60.71±12.39
Body mass index, mean±SD, kg/m^2^	27.70±5.44	28.06±5.62
Charlson comorbidity index, median (nterquartile)	1 (0 to 2)	1 (0 to 2)
Number of consultations, median (interquartile)	56 (0 to 111)	65 (32 to 113)
Number of referrals, median (interquartile)	0 (0 to 1)	1 (0 to 1)
Polypharmacy, median (interquartile)	7 (5 to 9)	6 (0 to 8)
Missing information: body mass index	16 226 (5.39)	23 555 (5.66)

ACL, anterior cruciate ligament; COPD, chronic obstructive pulmonary disease; COX2, cyclooxygenase-2; NSAIDS, non-steroidal anti-inflammatory drugs.

Multiple imputation using chained equations was applied to handle missing values, and the imputation model included all candidate predictors and outcome (online supplementary technical appendix).^34^

Based on the 15 509 THRs and a total of 73 predictor parameters and 18,289 TKRs and 79 predictor parameters, we had an effective sample size of 212 events per predictor parameter for the THR derivation cohort and 232 events per predictor parameter for the TKR derivation cohort, above the minimum requirement suggested by Peduzzi.^35^

In a sensitivity analysis, we assessed the models’ performances when including patients with a THR and TKR within the first 2 years after the index consultation (an exclusion criteria for the main analysis). In the other sensitivity analysis, we derived model coefficients by applying final predictors into patients with a THR and TKR within the first 2 years after the index consultation (an exclusion criteria for the main analysis).

We used Stata MP V.15.1 version for all statistical analyses. This study was conducted and reported in line with the transparent reporting of a multivariable prediction model for individual prognosis or diagnosis (TRIPOD) guidelines (online supplementary file 2).^30^

10.1136/annrheumdis-2018-213894.supp2 Supplementary data

## Results

### Study population

Table 1 summarises the baseline characteristics of the study populations, showing broadly similar characteristics between THR and TKR cohorts.

### Model development

Of 45 candidate categorical predictors of primary THR, 26 were excluded due to multivariable −1%≤PAR≤1% (online supplementary table S2). Of the remaining 19 categorical predictors and 6 continuous predictors considered for inclusion in the multivariable prediction model, 14 categorical predictors and 6 continuous predictors were retained after backward elimination (table 2). Previous hip injury recorded within 3 years prior to index consultation was a strong predictor of increased risk of future primary THR (adjusted subdistribution HR 1.54, 95% CI 1.40 to 1.69). Age at index consultation and body mass index (BMI) showed non-linear adjusted associations with THR, peaking at 75 years and 47 kg/m^2^ respectively (online supplementary figure S7).

**Table 2:** Adjusted subdistribution hazard ratios and final model coefficients

Predictor	Subdistribution hazard ratio (95 CI)	Beta coefficient
Final model for primary total hip replacement		
Gender: women vs men	1.00 (0.96 to 1.04)	0.002179
Smoking status		
Non-smoker/not recorded/ex-smoker	reference	
Light smoker	0.64 (0.54 to 0.75)	−0.446637
Moderate/heavy smoker	0.75 (0.70 to 0.80)	−0.283425
Drinking status		
Non-drinker/not recorded	reference	
Ex-drinker	1.01 (0.91 to 1.11)	0.008542
Light drinker	1.18 (1.13 to 1.24)	0.167673
Moderate drinker	1.36 (1.18 to 1.56)	0.304068
Heavy drinker	1.06 (0.83, 1.36)	0.062211
Diabetes mellitus: yes vs no	0.86 (0.81 to 0.91)	−0.154314
Mental disorders: yes vs no		
No/not recorded	reference	
Anxiety	0.85 (0.80 to 0.90)	−0.162867
Depression	0.85 (0.80 to 0.90)	−0.164488
Falls	0.85 (0.80, 0.91)	−0.157293
Previous hip injury: yes vs no	1.54 (1.40 to 1.69)	0.432446
Recorded diagnosis of joint-specific osteoarthritis (OA)		
No/not recorded	reference	
Knee OA	1.02 (0.64 to 1.61)	0.015210
Hand OA	0.21 (0.18 to 0.24)	−1.582914
Generalised OA	0.29 (0.25 to 0.33)	−1.242895
Other joint OA	0.23 (0.20 to 0.26)	−1.473753
Recorded diagnosis of non-specific OA: yes vs no	0.27 (0.26 to 0.28)	−1.312229
Analgesics		
No prescription	reference	
Weak combination opioids	0.93 (0.89 to 0.97)	−0.072075
Moderate combination opioids	1.00 (0.85 to 1.19)	0.002597
Strong/very strong combination opioids	1.06 (0.87 to 1.29)	0.056441
Antidepressant: yes vs no	0.96 (0.92 to 1.01)	−0.036091
Topical NSAIDS		
No prescription	reference	
NSAIDS	0.77 (0.73 to 0.80)	−0.266746
Other	0.78 (0.65 to 0.94)	−0.247566
NSAIDS/COX2		
No prescription	reference	
NSAIDS	1.06 (1.02 to 1.10)	0.056447
COX2	1.18 (1.10 to 1.26)	0.164306
Hormone treatment: yes vs no	0.96 (0.92 to 1.01)	−0.039146
(Age/10)^3	–	0.056927
(Age/10)^3*ln(age/10)	–	−0.024913
(Body mass index (BMI)/10)^2	–	0.137871
(BMI/10)^3	–	−0.024987
((Charlson comorbidity index+1)/10)^−2	–	0.001583
((Charlson comorbidity index+1)/10)^2	–	−1.318626
((Number of referrals+1)/10)^−2	–	−0.015522
((Number of referrals+1)/10)^−2*ln((number of referrals+1)/10)	–	−0.005468
((Number of consultations+1)/1000)^−0.5	–	−0.171643
((Number of consultations+1)/1000)^−0.5*ln((number of consultations+1)/1000)	–	−0.017409
((Number of BNF chapters+1)/10)^−2	–	0.110533
((Number of BNF chapters+1)/10)^−2*ln((number of BNF chapters+1)/10)	–	0.046402
Final model for primary total knee replacement		
Gender: women vs men	0.87 (0.85 to 0.90)	−0.136204
Ethnicity		
White	reference	–
Other ethnicity group	0.90 (0.75 to 1.08)	−0.105427
Not recorded	1.04 (1.01 to 1.08)	0.042038
Smoking status		
Non-smoker/not recorded/ex-smoker	reference	–
Light smoker	0.75 (0.64 to 0.89)	−0.281159
Moderate/heavy smoker	0.75 (0.71 to 0.80)	−0.281584
Drinking status		
Non-drinker/not recorded	reference	–
Ex-drinker	1.13 (1.09 to 1.50)	0.126359
Light drinker	1.21 (1.07 to 1.38)	0.192886
Moderate drinker/heavy drinker	1.34 (1.19 to 1.50)	0.289711
Asthma, yes vs no	1.07 (1.03 to 1.12)	0.070898
COPD, yes vs no	0.71 (0.66 to 0.77)	−0.341224
Diabetes mellitus: yes vs no	0.88 (0.84 to 0.93)	−0.126142
Mental disorders: yes vs no		
Anxiety	0.76 (0.73 to 0.80)	−0.268390
Depression	0.85 (0.81, 0.89)	−0.164173
Previous knee injury: yes vs no	1.29 (1.24 to 1.35)	0.256978
Recorded diagnosis of joint-specific OA		
No/not recorded	reference	–
Hip OA	0.59 (0.55 to 0.63)	−0.528500
Hand OA	0.61 (0.56 to 0.68)	−0.486247
Generalised OA/	0.76 (0.69 to 0.83)	−0.278839
Other joint OA	0.74 (0.69 to 0.80)	−0.296179
Recorded diagnosis of non-specific OA: yes vs no	1.17 (1.12 to 1.22)	0.159204
Low back pain: yes vs no	0.87 (0.84 to 0.90)	−0.142815
Hypertension: yes vs no	0.96 (0.93 to 0.99)	−0.043010
Joint injection: yes vs no	1.66 (1.60 to 1.72)	0.504619
Knee arthroscopy: yes vs no	14.47 (13.95 to 15.02)	2.672150
Antidepressant: yes vs no	0.95 (0.91 to 0.98)	−0.054225
Analgesics		
No prescription	reference	–
Weak combination opioids	1.33 (1.27 to 1.39)	0.286170
Moderate combination opioids	1.37 (1.22 to 1.54)	0.318097
Strong/very strong combination opioids	1.59 (1.45 to 1.75)	0.465081
Topical NSAIDS		
No prescription	reference	–
NSAIDS	0.93 (0.90 to 0.96)	−0.074381
Other	1.17 (1.08 to 1.28)	0.160424
NSAIDS/COX2		
No prescription	reference	–
NSAIDS	1.41 (1.35 to 1.48)	0.343065
COX2	1.27 (1.19 to 1.36)	0.238566
(Age/10)^3	–	0.029250
(Age/10)^3*ln(age/10)	–	−0.013246
(BMI/10)^2	–	0.280906
(BMI/10)^3	–	−0.047226
((Charlson comorbidity index+1)/10)^−2	–	0.002381
((Charlson comorbidity index+1)/10)^2	–	−1.442397
((Number of referrals+1)/10)^−2	–	−0.010564
((Number of referrals+1)/10)^−2*ln((number of referrals+1)/10)	–	−0.002929
((Number of consultations+1)/1000)^−0.5	–	−0.542288
((Number of consultations+1)/1000)^−0.5*ln((number of consultations+1)/1000)	–	−0.073453

BNF, British National Formulary; COPD, chronic obstructive pulmonary disease; COX2, cyclooxygenase-2; NSAIDS, steroidal anti-inflammatory drugs.

Of 53 candidate categorical predictors of primary TKR, 20 were excluded due to multivariable −1%≤PAR≤1% (online supplementary table S3). Of the remaining 33 categorical predictors and 6 continuous predictors entered into the multivariable prediction model, 19 categorical predictors and 5 continuous predictors were retained after backward elimination (table 2). Oral NSAID and opioid analgesic prescriptions, intra-articular injections and previous arthroscopic knee surgery in the 3 years prior to index consultation were strong predictors of increased risk of future primary TKR. Age and BMI showed non-linear adjusted associations, peaking at 70 years and 40 kg/m^2^ respectively (online supplementary figure S8).

### Apparent predictive performance of the models

Our final THR prediction model was able to discriminate between patients with and without a primary THR with a C-statistic of 0.73 (95% CI 0.72 to 0.73); our final TKR prediction model was also able to discriminate between patients with and without TKR with a C-statistic of 0.79 (0.78 to 0.79) over the 10-year follow-up period. The calibration slope was 1.00 (0.98 to 1.02) and 1.00 (0.99 to 1.01) for THR and THR, respectively, as we would expect.

### Internal–external cross-validation

**Figure 1:**
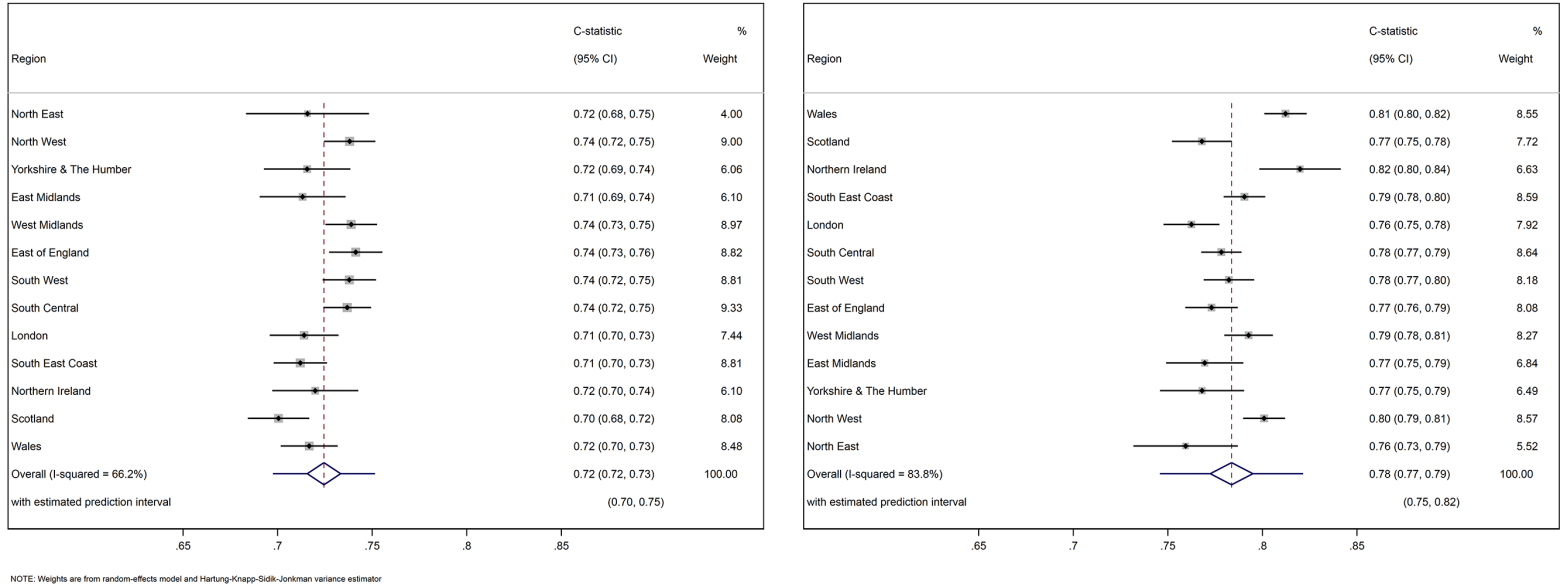
C-statistics in 13 validation cohorts and the overall estimation across validation cohorts. The left panel is for THR model and the right is for TKR model.

The internal-external cross-validation revealed that the C-statistic was similar in each of the 13 geographical regions, ranging from 0.70 (0.68 to 0.72) to 0.74 (0.73 to 0.75) for the THR model (figure 1 left panel) and between 0.76 (0.73 to 0.79) and 0.82 (0.80 to 0.84) for the TKR model (figure 1 right panel). After meta-analysis, the summary C-statistic was 0.72 (0.72 to 0.73) for the THR model and 0.78 (0.77 to 0.80) for the TKR model. Based on the 95% prediction intervals, if the models were applied in a new (but similar) setting, we would expect the C-statistic to be between 0.70 and 0.75 for the THR model and between 0.75 and 0.82 for the TKR model.

**Figure 2:**
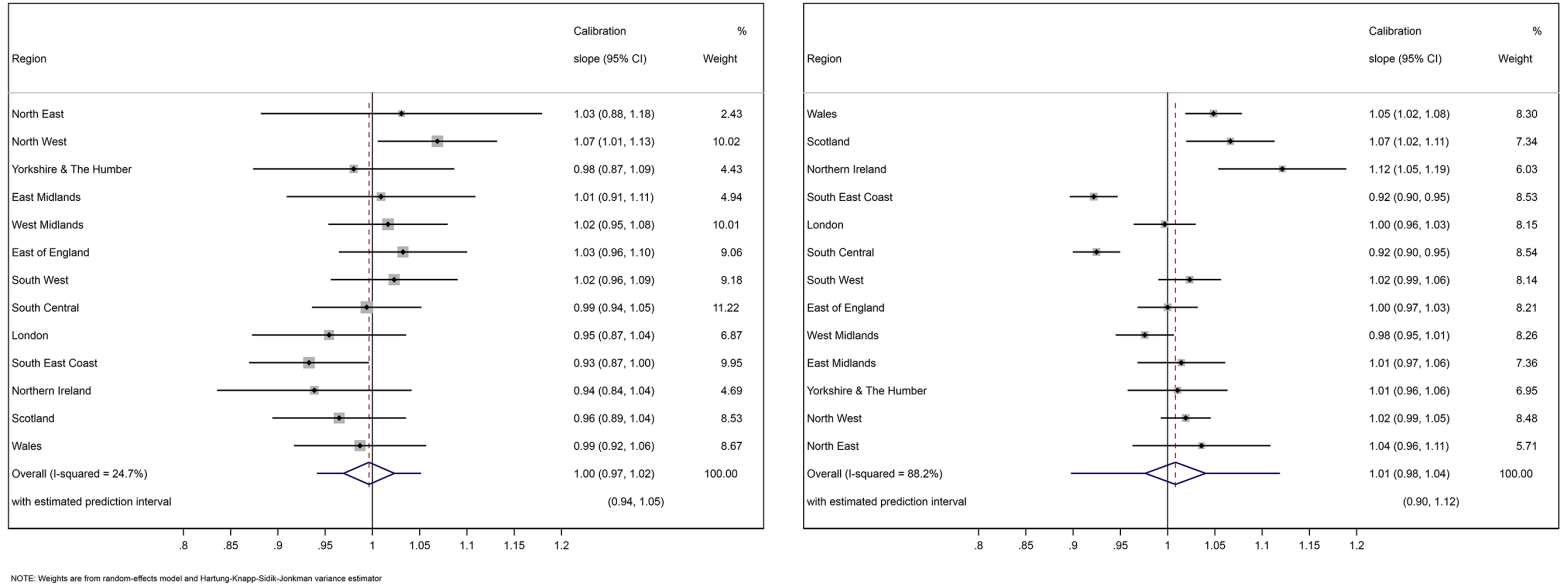
Calibration slope in 13 validation cohorts and the overall estimation across validation cohorts. The left panel is for THR model and the right is for TKR model.

The calibration slope was also similar across the 13 regions, ranging between 0.93 (0.87 to 1.00) and 1.07 (1.01 to 1.13) for THR model (figure 2 left panel) and between 0.92 (0.90 to 0.95) and 1.12 (1.05 to 1.19) for TKR model (figure 2 right panel). After meta-analysis, the summary calibration slope was 1.00 (0.97 to 1.02) for the THR model and 1.01 (0.98 to 1.04) for the TKR model. If the models were applied in a new (but similar) setting, we would expect the calibration slope to be between 0.94 and 1.05 for the THR model and between 0.98 and 1.12 for the TKR model.

**Figure 3:**
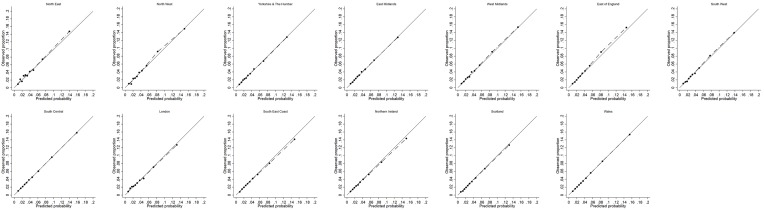
Assessment of calibration for model predicting 10-year risk of primary total hip replacement in validation cohorts.

**Figure 4:**
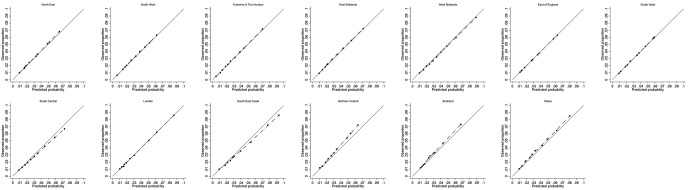
Assessment of calibration for model predicting 10-year risk of primary total knee replacement in validation cohorts.

The calibration plot in each validation cohort was presented for one imputed dataset, but comparable to the calibration plots in the other imputations (figure 3 for THR and in figure 4 for TKR). Good agreement between observed and predicted risks was observed in each geographical region cohort for THR. For those at highest risk of TKR (>10th decile), the observed risk was slightly higher than the predicted risk from the model in Northern Ireland, Scotland and Wales. We hypothesised that this slight miscalibration could result from systematic differences between these devolved nations and the English regions in the entry year or follow-up duration. However, on inspection, this was not the case (online supplementary figures S9 and S10).

The sensitivity analysis including patients with early outcomes (THR/TKR within 2 years of index consultation) gave similar levels of discrimination and calibration (online supplementary figure S11 and table S4). Similar model coefficients were derived by applying final predictors into patients with early outcomes (THR/TKR within 2 years of index consultation) (online supplementary table S5).

### Clinical examples

Online supplementary tables S6 and S7 give clinical examples of the application of THR and TKR risk prediction models to predict 10-year risk of THR and TKR, with examples chosen to illustrate ‘low’ and ‘high’ risk.

## Discussion

We have developed and validated two algorithms to predict the absolute risk of future primary THR and TKR in patients who newly present to UK general practice with hip pain/osteoarthritis and knee pain/osteoarthritis. Internal–external cross-validation showed consistently good calibration when models are applied to the different geographical regions, and the models have good discrimination with C-statistics of greater than 0.70 for both models. To our knowledge, these are the first such risk prediction tools, for primary THR and TKR in osteoarthritis developed in large-scale cohort data.

### Strengths and limitations of study

Our study was based on a large, representative and contemporary UK population with data obtained from a validated research database.^19^ Risk prediction tools relying on routinely collected primary care data are more readily implementable in primary care practice^10^, and this was an important motivation for our study design. Potential limitations include missing predictor data and known predictors that are not measured or recorded in the primary care EHR. Around 5% of cohort participants did not have a recorded value for BMI in the 3 years prior to index pain/osteoarthritis consultation, but we found little difference in findings between complete dataset and multiple imputed datasets. We assumed no consultation record of a morbidity or prescription meant, there had been no such event within primary care. Although it is a fairly standard approach to use the most recent record for time-varying exposures that are likely to be generally stable, this approach might still be conservative (ie, underestimate the exposure–outcome association) to the extent that it misclassifies the exposure level relevant to the outcome (eg, lifetime cumulative exposure to smoking).

Primary THR and TKR are complex, multiply determined outcomes and can be considered as a composite measure of osteoarthritis progression, since these procedures are indicated for a combination of pain, functional disability, impact on quality of life, radiological changes and failed conservative treatment.^36^ Joint replacement is an important outcome of osteoarthritis, and this is reflected in its role when judging the validity of imaging-related primary endpoints for clinical trials of structure-modifying drugs.^37^ However, it is important to recognise that a proportion of individuals with progressive OA may not be offered, or accept, TKR/THR. The receipt of TKR/THR can also reflect extraneous factors such as patient age, sex, ethnicity, willingness to undergo surgery, comorbidity, patients’ needs, patients’ coping skills, physician effect and prevailing supply-side factors.^38^ We found adjusted rates of THR and TKR were lower given the following patient characteristics at or before index hip/knee consultation: age over 80–85 years, non-white ethnicity,^39^ higher levels of comorbidity (including diagnosed mental health disorder, generalised OA and low back pain) and very high levels of obesity. Osteoarthritis progression in the context of these factors will be underestimated by risk algorithms based on the outcome of receipt of primary joint replacement.

A strength of our study was the comprehensive identification of candidate predictors from a variety of sources including a novel hypothesis-free ‘ReWAS’ study with replication in an independent primary care EHR dataset. This latter technique yielded a small number of candidate predictors not previously reported (eg, arthroscopy). ReWAS also confirmed known prognostic factors or suggested prognostic factors that were most likely proxy markers for known predictors not obtainable from the EHR (eg, analgesic prescriptions as a proxy for pain severity). All such ‘hits’ had to be judged clinically relevant by review panel of clinicians and lay member in order to be included in the modelling stage. Unsurprisingly, many candidate predictors of future primary THR or TKR previously identified in the literature were not routinely available within the primary care record. These included multi-item patient-reported measures of pain severity,^40 41^ structural disease markers from plain X-rays or MRI^40 42^ and measures of occupational and leisure time physical activity.^43–46^ It is not known if their inclusion would significantly improve model performance.

### Comparison with other studies

The majority of relevant previous studies have focused on one or more potential causal exposures for future joint replacement. We identified only three small studies that had previously derived and reported a multivariable prediction model for total hip or knee replacement based mainly on patient-reported and imaging variables.^40 41 47^ Although the overall performance of the prognostic model is of primary importance, the direction and magnitude of association between some of the included predictors and outcome deserves comment. It must be recognised though that these associations are not intended to be, and cannot be interpreted as, valid estimates of causal effect (total, direct or indirect) on primary hip/knee replacement: they are chosen for their informativeness in predicting primary THR/TKR. They may or may not be causal or reversible; all associations were conditioned on having an index consultation for hip or knee osteoarthritis/pain; each coefficient was adjusted for all covariates in the model, but the minimally sufficient set of covariates needed to adjust for confounding would likely differ for each (the ‘table 2 fallacy’^48^). With these concerns in mind, we note that adjusted rates of THR and TKR were lower among moderate/heavy current smokers^49^ and higher among those with a previous injury.^50^ Prior arthroscopic knee surgery was strongly associated with future TKR, an association which may include a very small direct causal effect^51^ but which otherwise we interpret as reflecting a mixture of disease severity, risk of future progression and willingness to undergo a surgical procedure for the knee.

### Implications

Our newly developed risk algorithms could have important applications in clinical practice by helping direct annual monitoring, intensive non-surgical care and timely assessment and discussion of the need for surgical referral to those most at risk of progression. The algorithms can specifically identify the individuals who, in the context of current healthcare policies and resources, are at higher risk of future joint replacement, and therefore can be targeted for individual care ranging from earlier surgery to non-invasive care that might postpone the need for surgery. The hypothetical higher risk individual illustrated in online supplementary table S6 and S7 might, for instance, be targeted for a programme of more intensive multimodal therapy including graded supervised exercise and supported weight loss. The algorithm also uses future joint replacement as a proxy for future progression of osteoarthritis, and therefore potentially attempting to identify individuals more broadly who can be targeted for more intensive monitoring and interventions that might prevent such future progressions and severity regardless of whether they would actually have had a joint replacement. For each of these clinical activities, a more targeted approach based on risk of progression may help. Monitoring of patients with osteoarthritis for progression of symptoms and impact is regarded as an important aspect of quality of care,^52^ but current The National Institute for Health and Care Excellence guidance would result in this being applied to a very large number of patients.^53^ Consideration for joint replacement should only be made after proper conservative care.^3^ While consistent evidence supports the effectiveness for knee OA of supervised, individually tailored exercise programmes progressed over several visits,^6^ many patients do not receive this^52^ partly due to limited physiotherapy resource^8^ and lack of referral.^7^ For many patients, joint replacement will still be the most cost-effective intervention and an earlier recognition of patients’ risk of future joint replacement may facilitate more timely assessment and discussion of appropriateness for referral. However, we caution against over-reliance on these risk algorithms, particularly for individuals whose characteristics mean that they will not be candidates for surgery despite experiencing progressive disease, and against their crude application to ration what are highly cost-effective procedures of primary THR and TKR for osteoarthritis.

### Conclusions

We have developed and validated two new risk prediction equations to quantify the absolute risks of primary THR and TKR in patients’ newly presenting with hip pain/OA or knee pain/OA in the primary care setting. The models have the advantage of being based on information routinely available in UK primary care EHR, making them potentially implementable for automatic risk calculation in electronic medical record software. They can be used to identify patients at high risk of end-stage OA for further assessments and intensive non-surgical intervention. The algorithms are readily amenable to further external validation in many developed countries that have routine records available for research. Further research is warranted to evaluate the clinical outcomes and cost effectiveness of using these risk equations in primary care.
