# Supplementary material for: Development and validation of prediction models to estimate risk of primary total hip and knee replacements using data from the UK: two prospective open cohorts using the UK Clinical Practice Research Datalink
Source: Ann Rheum Dis. 2018 Oct 18;78(1):91–9. doi: 10.1136/annrheumdis-2018-213894 (PMC6317440; doi:10.1136/annrheumdis-2018-213894)
Supplement: Supplementary data [file annrheumdis-2018-213894supp001.docx]

**Online Supplemental File 1**

**Supplementary Technical Appendix**

*The definition and the selection of study population*

We selected patients with a recorded consultation for hip pain/osteoarthritis or knee pain/osteoarthritis pain between 1 January 1992 and 31 December 2015 and who had complete registration and no recorded hip pain/osteoarthritis or knee pain/osteoarthritis consultations in the 3 years before their index consultation (the first consultation for hip or knee pain/osteoarthritis recorded in the study period, reflecting a first or new episode). The entry date for each patient was the date of index consultation.

Consultations for hip pain/osteoarthritis and knee pain/osteoarthritis were defined using morbidity (“Read”) codes for diagnosed osteoarthritis and hip or knee pain codes likely to represent osteoarthritis previously determined by six academic general practitioners with an interest in musculoskeletal conditions [22]. Read codes are used to record morbidity in UK primary care and the codes used are available from [www.keele.ac.uk/mrr](http://www.keele.ac.uk/mrr).

*Defining primary total hip and knee replacement*

We used the earliest recorded date of the outcome after the index consultation. To reduce the potential for reverse causality (e.g. the act of referring for joint replacement led to recording of osteoarthritis), we excluded patients with hip or knee replacements within 2 years of the index consultation (initial descriptive (Kaplan-Meier) analysis identified a much higher risk of TKR / THR in the first 2 years that is likely to be attributable to reverse causality), and those whose records were censored (died, de-registration with practice, or last upload of computerised data) during this 2-year period. Among patients with both hip and knee replacements, their hip replacements were defined as outcomes in the hip model and knee replacements were defined as outcomes in the knee model.

*Systematic review and source of conventional risk factors*

Briefly, we searched Medline, Embase, Allied and Complementary, Cumulative Index to Nursing and Allied Health Literature and Web of Science™ core collection up to December 2014 for all studies investigating risk factors for THR and TKR. 35 studies were undertaken in 22 unique datasets from 12 different countries. Of the 35 studies identified, 16 rated as high quality were used in the best evidence synthesis, in which there were 21 possible predictors for TKR and 28 possible predictors for THR identified and include in the comprehensive list.

35 conventional predictors used across QResearches were also extracted and included in the comprehensive list.

*ReWAS study*

96,450 and 165,413 incident THR and TKR cases between 1992 - 2015 were identified from the CPRD using 3-year run-in period. An age-, gender- and practice-matched control with ≥1 consultations in the past 3 years was selected for each case using risk-set sampling [^1^]. For ReWAS, firstly, we identified all third-level Read codes and third-level sections within the British National Formulary (BNF) which had been recorded in ≥1% of cases in the 3 years prior to date of total joint replacement as potential prognostic factors (n=6,109 and 325 respectively); secondly, we used conditional logistic regression to estimate their association with outcome, with ‘hits’ defined as those factors with a population attributable risk (PAR) ≥ 1% or ≤ -1% (**S3 Fig upper panel:** 3^rd^ level read codes for TKR **and S4 Fig upper panel:** 3^rd^ level read code for THR; **S5 Fig upper panel:** BNF sections for TKR **and S6 Fig upper panel:** BNF section for THR) and with significant Bonferroni-corrected P-values (**S3 Fig lower panel:** 3^rd^ level read codes for TKR and **S4 Fig lower panel:** 3^rd^ level read code for THR; **S5 Fig lower panel**: BNF sections for TKR and **S6 Fig lower panel:** BNF section for THR). Finally, we repeated the ReWAS method in a case-control study with 874 incident knee/hip replacement cases and 4,370 matched controls, derived from a regional dataset - Consultations in Primary Care Archive (CiPCA). We identified 36 ‘hits’, i.e. potential prognostic factors recorded in ≥ 1% of cases, and having PAR ≥ 1% /≤ -1% and P-value < 1.539×10-4 (32 for THR; 33 for TKR (29 associated with both outcomes)).

*Definition of predictors*

Comorbidities (i.e. anxiety or depression), process of care, and prescription predictors identified from REWAS were defined based on their corresponding code at the 3rd level of Read codes hierarchy or BNF section. Potential predictors identified from the systematic review and the QResearch algorithms were defined using code lists used in previous Centre studies developed though consensus of expert GP and EHR researchers, or if these did not exist, by modifying code lists used in external studies and / or consensus of 7 GP and EHR researchers.

Smoking status was defined as non-smoker, ex-smoker, light smoker (1-9 cigarettes/day), moderate smoker (10-19 cigarettes/day), and heavy smoker (≥20 cigarettes/day); drinking status was defined as non-drinker, ex-drinker, light drinker (1-2 unit/day), moderate drinker (3-6 unit/day) and heavy drinker (≥ 7 unit/day); physical activity status was defined as no physical activity (General practice physical activity questionnaire physical activity index: inactive), taking light physical activity (General practice physical activity questionnaire physical activity index: moderately inactive), taking moderate physical activity (General practice physical activity questionnaire physical activity index: moderately active) and taking heavy physical activity (General practice physical activity questionnaire physical activity index: active).

*Statistical analysis for model derivation*

For the 45 categorical candidate predictors in the THR derivation cohort and 53 categorical candidate predictors in the TKR derivation cohort, we first fitted a full multivariable Fine-Gray subdistribution hazard model, accounting for the competing risk of death (including all candidate predictors) to calculate the adjusted hazard ratio for receiving a THR/TKR. Candidate predictors with population attributable risk (PAR) >1% (or <-1%) were taken forward to the next stage of model development in which multivariable Fine-Gray models were fitted to the remaining categorical predictors, and the 6 continuous predictors. A multivariable fractional polynomial approach was used, in which fractional polynomial terms were considered to flexibly model the 6 continuous predictors while at the same time using backward elimination (p>0.1 based on log likelihood) to exclude seemingly unimportant predictors from the model.

*Model performance evaluation*

We assessed the performance of each model in terms of both discrimination and calibration. Discrimination measures the model’s ability to distinguish between individuals that will and will not have a THR/TKR, and was measured using Harrell’s C-statistic (0.50 represents no discrimination, 1.00 perfect discrimination) [30]. Calibration measures the agreement between the observed risks and the model’s predicted risks, and was evaluated using the calibration slope (a value of 1.00 is ideal) [30-32] over the 10-years of follow-up. Calibration plots were also produced, in which patients were classified into 10 risk groups based on their predicted probability of the outcome.

*Internal-external cross-validation (IECV) approach*

Using this approach, a single cycle of IECV involves separating the data into a development cohort and validation cohort, with 12 of the 13 geographical regions forming the development cohort and reserving the other region for validation. The model development strategy (PAR elimination + backward elimination) was applied to the development cohort and the derived model was then validated in the reserved validation cohort. This process was repeated multiple times, each time reserving a different region for validation, therefore there were 13 cycles and each geographical region was used once for validation (see table below).

| Cycle of IECV | Development cohort | Validation cohort |
| --- | --- | --- |
| 1 | Regions 2-13 | Region 1 |
| 2 | Regions 1, 3-13 | Region 2 |
| 3 | Regions 1, 2, 4-13 | Region 3 |
| … | … | … |
| 13 | Regions 1-12 | Region 13 |

Internal validation aims to assess the reproducibility of the model, i.e. evaluates the modelling process. External validation aims to assess the generalisability of the model (to different regions in this case). Therefore the IECV process can be used to give more realistic estimates of model performance (than apparent performance) and also reveal how the developed models are likely to perform in new data, independent to that used for model development.

Performance statistics were also summarised across regions using a random-effects meta-analysis, reporting the average performance statistic with 95% confidence interval (derived using the Hartung-Knapp-Sidik-Jonkman variance correction) and 95% prediction interval.

*Handling of missing data*

Our derivation cohorts had missing information on body mass index (5.39% for THR cohort and 5.26% for TKR cohort). For missing values in the categorical variables (ethnicity, smoking status, drinking status, mental health disorder, and joint specific osteoarthritis), a ‘not recorded’ category was introduced and combined with the reference category for each variable. We used multiple imputation within each derivation cohort (THR and TKR cohorts) prior to model development and validation. Multiple imputation using chained equations (MICE) was applied, and the imputation model included all candidate predictors and outcome. We created 5 imputed datasets, and final model estimates were obtained by combining estimates across all imputed datasets using Rubin’s rules [36]. In the validation, performance statistics were also combined across imputations using Rubin’s rules. However, calibration plots were produced for each imputed dataset and presented for one imputed dataset if representative of what was seen in the other imputations.

**Supplementary figure S1**. Workflow chart of the selection of participants for total knee replacement model


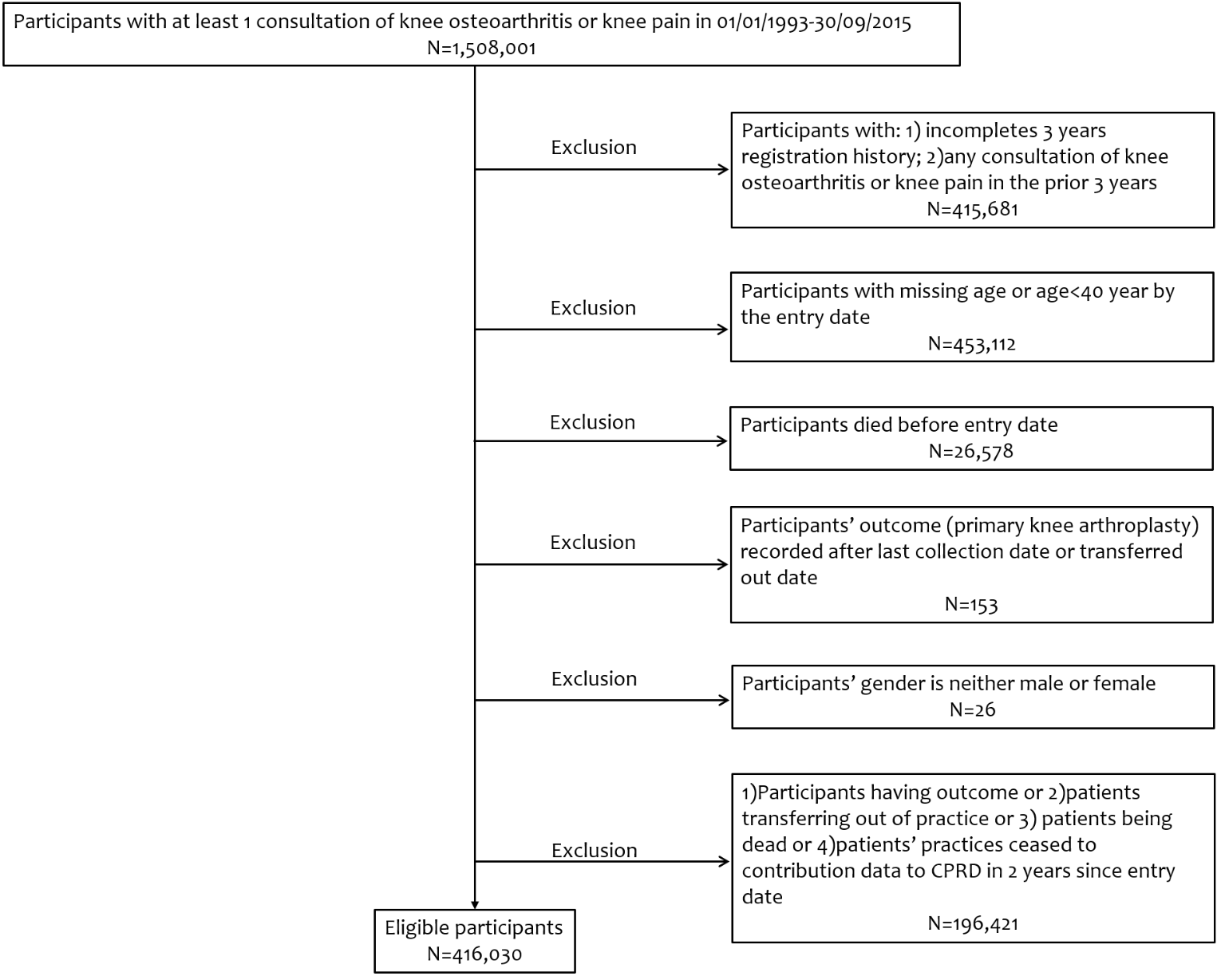


**Supplementary figure S2**. Workflow chart of the selection of participants for total hip replacement model


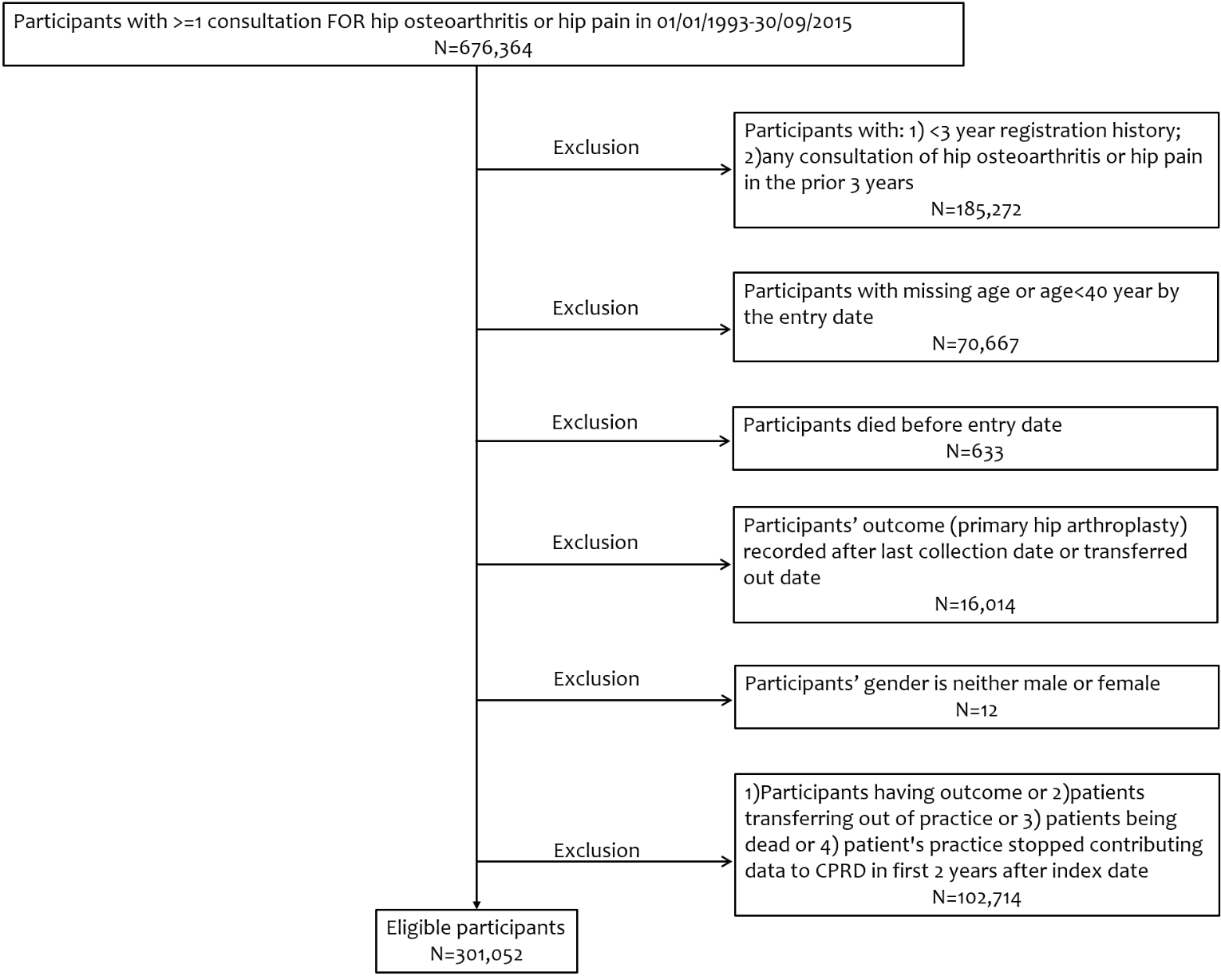


**Supplementary figure S3**. Population attributable risk for association between each third level Read code and total knee replacement: findings from population based case-control study

**High-resolution figure** could be found at: [**https://doi.org/10.6084/m9.figshare.5249041.v1**](https://doi.org/10.6084/m9.figshare.5249041.v1)


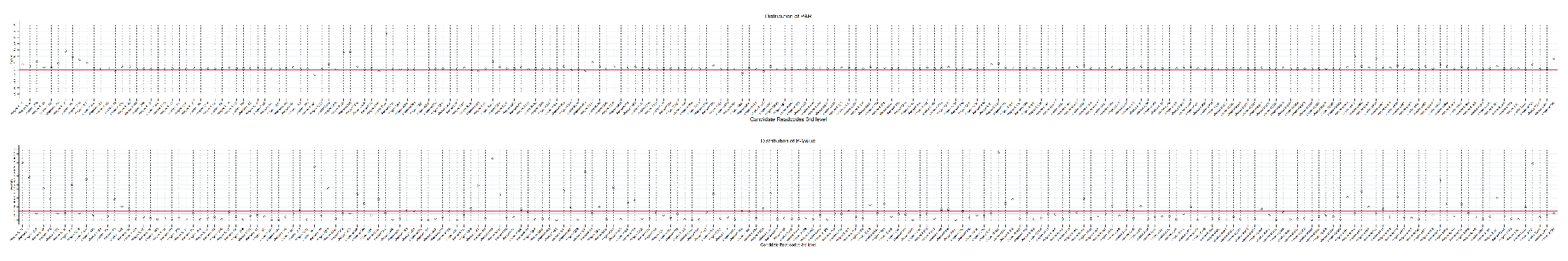


**Supplementary figure S4**. Population attributable risk for association between each third level Read code and total hip replacement: findings from population based case-control study

**High-resolution figure** could be found at: [**https://doi.org/10.6084/m9.figshare.5249047.v1**](https://doi.org/10.6084/m9.figshare.5249047.v1)


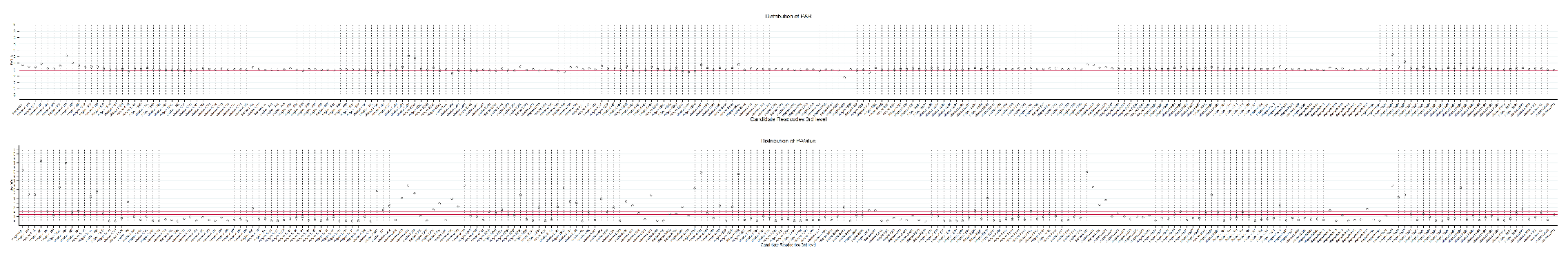


**Supplementary figure S5**. Population attributable risk for association between each BNF section and total knee replacement: findings from population based case-control study

**High-resolution figure** could be found at: [**https://doi.org/10.6084/m9.figshare.5249059.v1**](https://doi.org/10.6084/m9.figshare.5249059.v1)


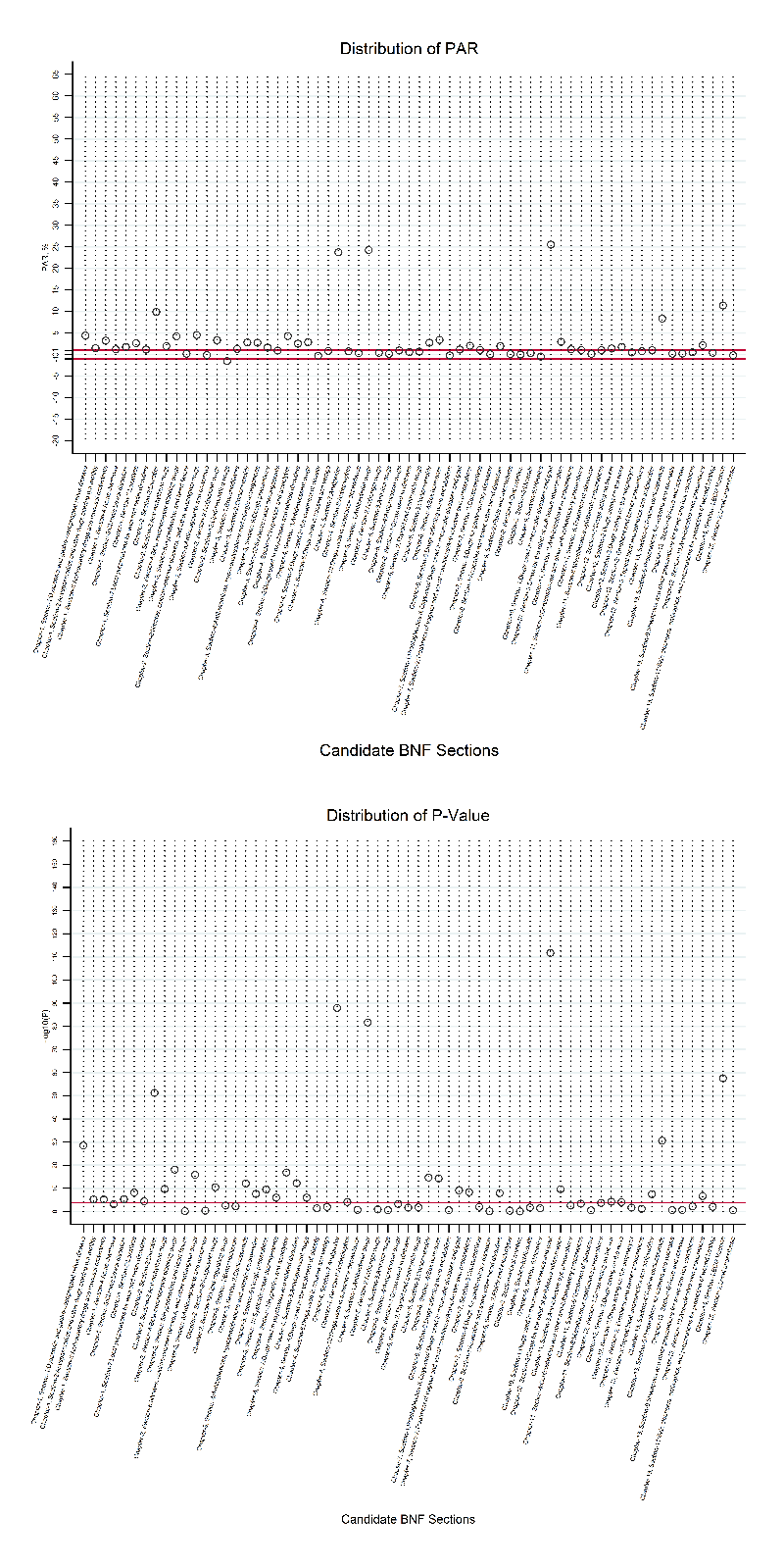


**Supplementary figure S6**. Population attributable risk for association between each BNF section and total hip replacement: findings from population based case-control study

**High-resolution figure** could be found at: [**https://doi.org/10.6084/m9.figshare.5249068.v1**](https://doi.org/10.6084/m9.figshare.5249068.v1)


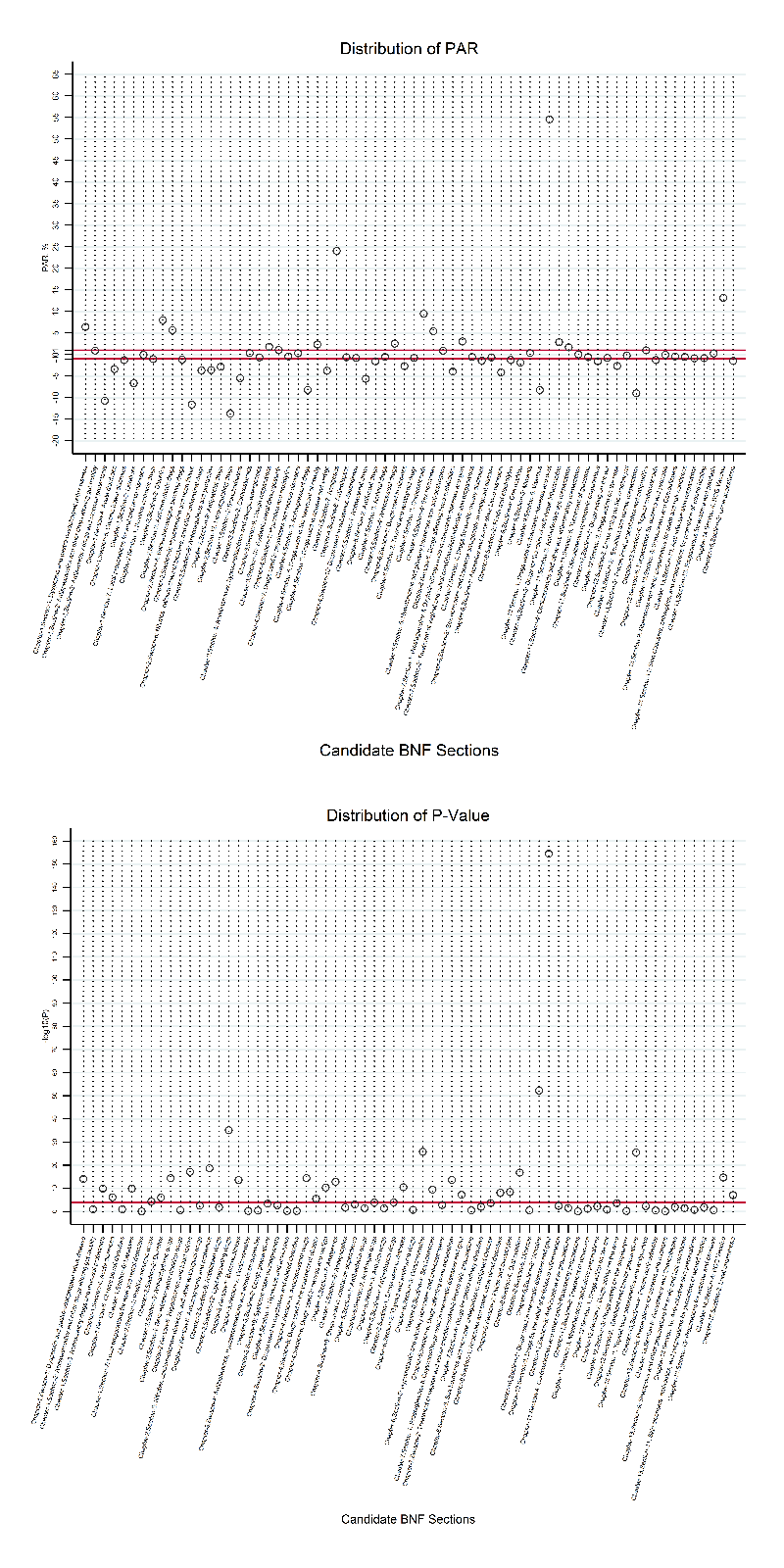


**Supplementary Figure S7. Adjusted subdistribution hazard ratios for primary total hip replacement by age, body mass index, Charlson comorbidity index, consultation counts, referrals, and polypharmacy (BNF chapters) in THR derivation cohort**
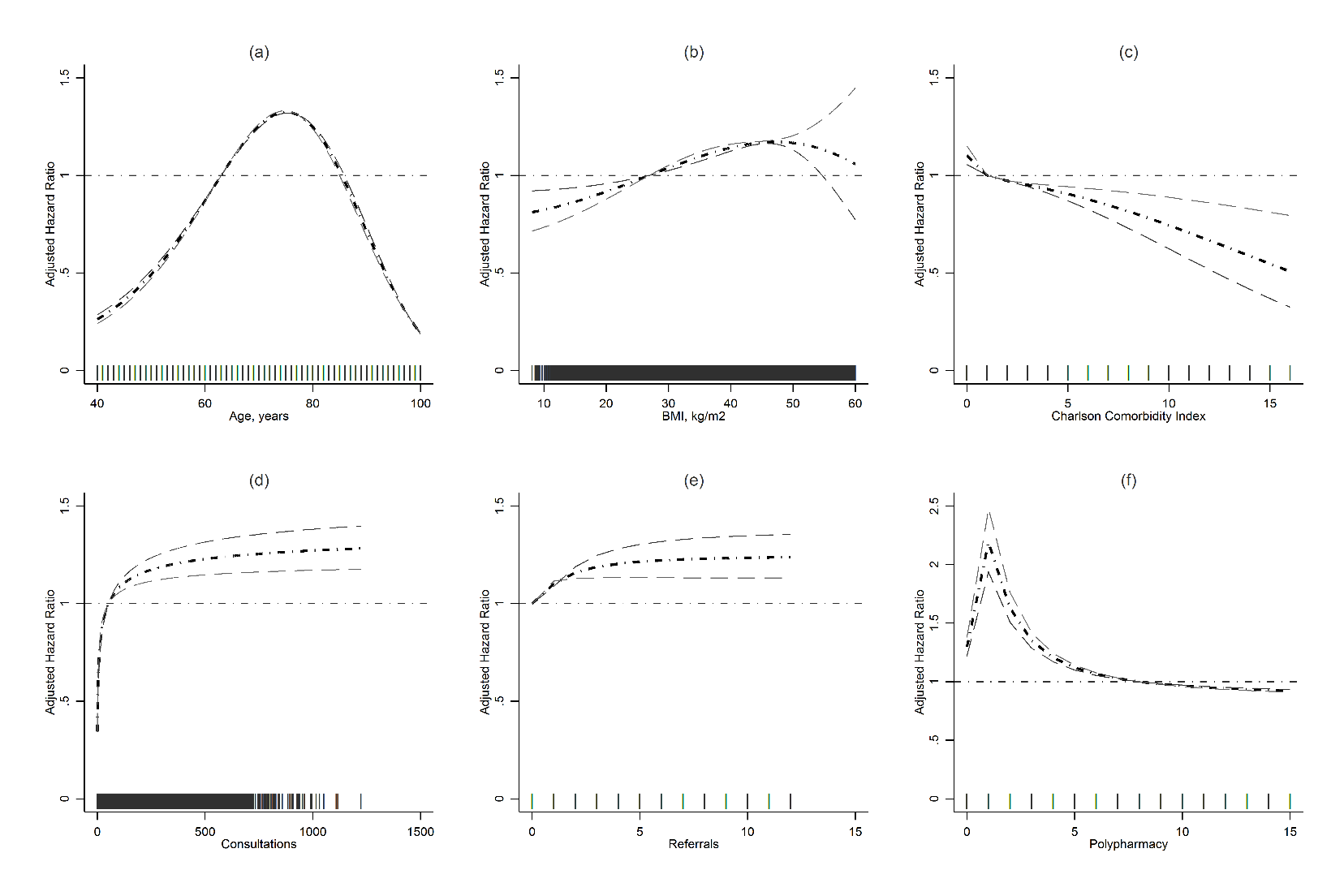


**Supplementary Figure S8. Adjusted subdistribution hazard ratios for primary total knee replacement by age, body mass index, Charlson comorbidity index, consultation counts, referrals in TKR derivation cohort**

*
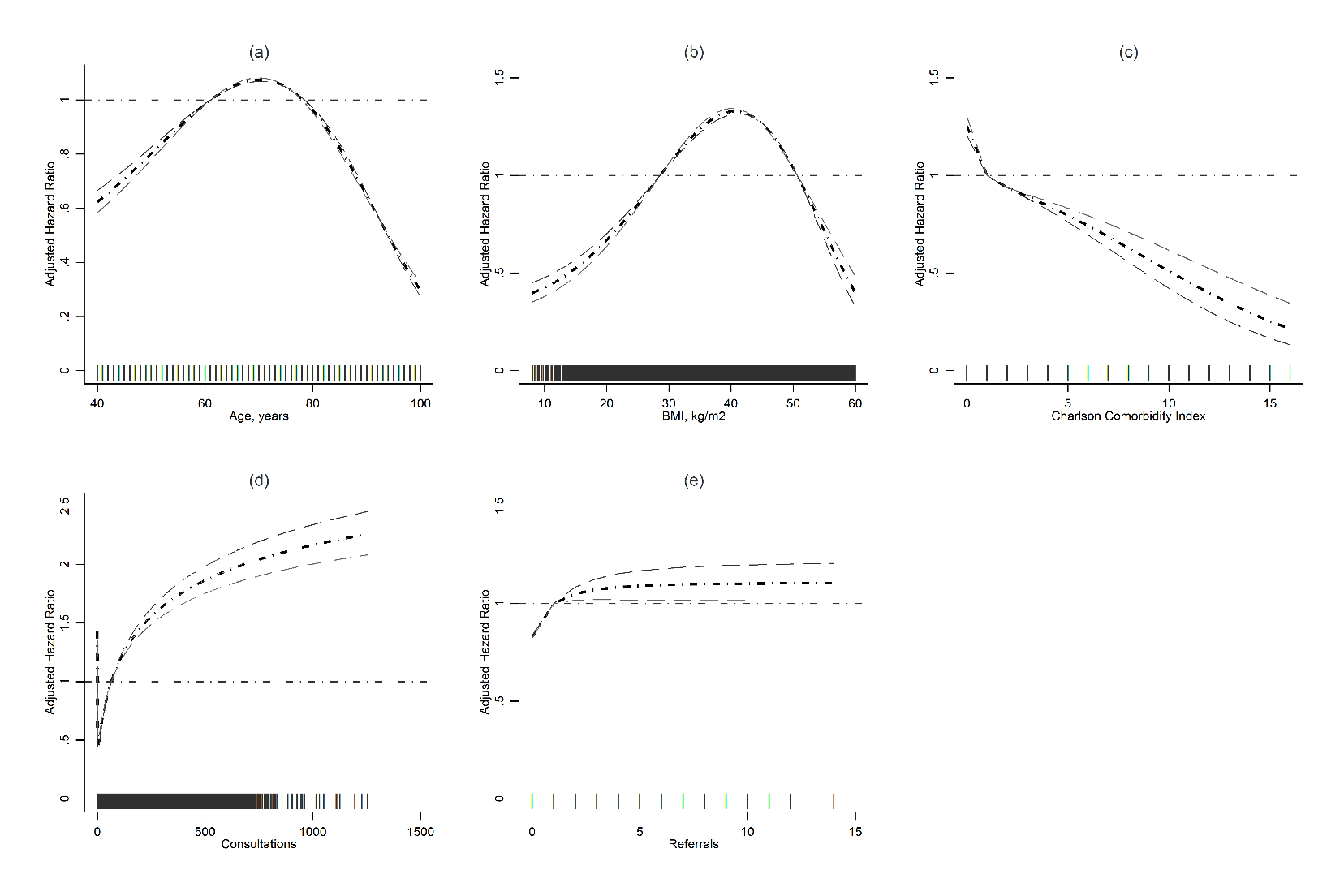
*

**Supplementary Figure S9**. Distribution of entry year by regions in TKR cohort

Dash-line indicates the distribution in the whole TKR cohort; the solid line indicates the distribution in the regional cohort.


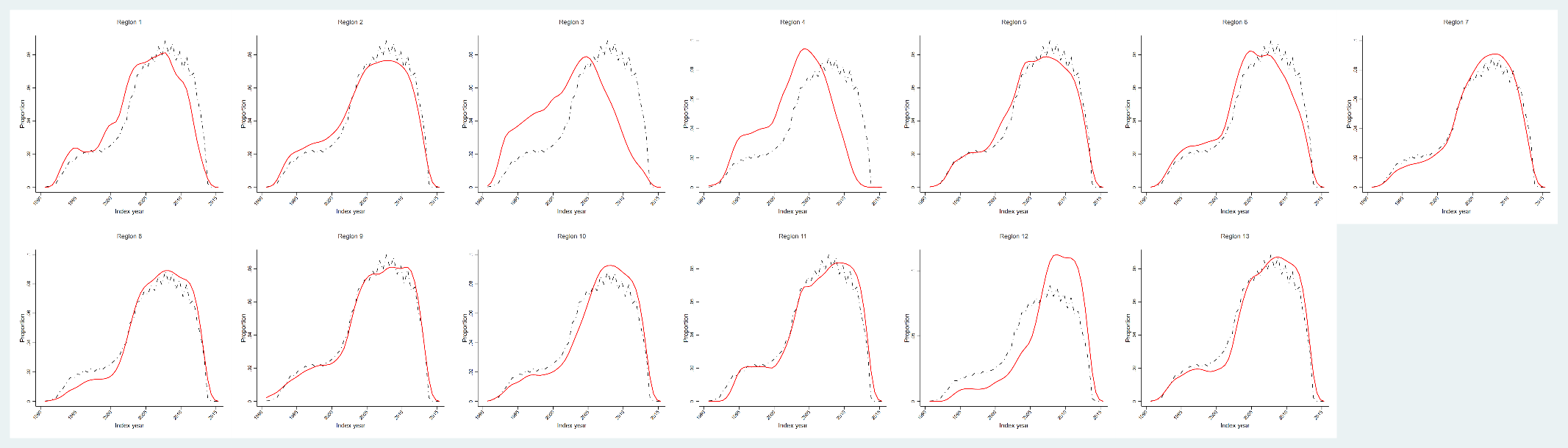


**Supplementary Figure S10**. Distribution of follow-up time by regions in TKR cohort

Dash-line indicates the distribution in the whole TKR cohort; the solid line indicates the distribution in the regional cohort.


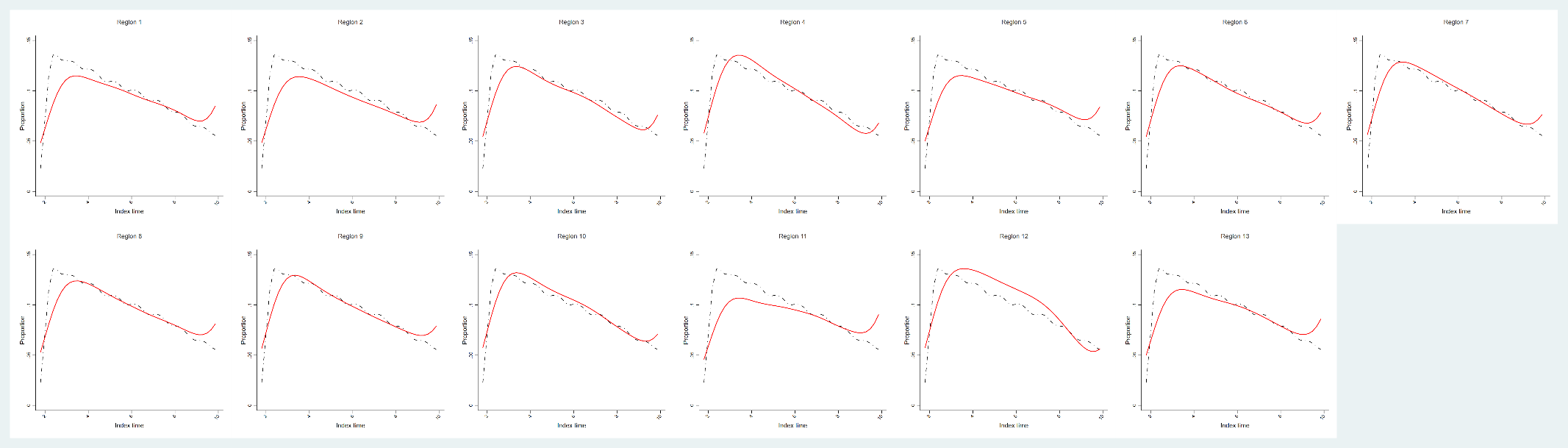


**Supplementary Figure S11**. Sensitivity analysis: apply risk algorithms in the sample incorporating patients with early (within 2 years since entry date) outcomes The calibration plot in each validation cohort was presented for one imputed dataset, but comparable to the calibration plots in the other imputations (**Left** for THR and in **Right** for TKR).


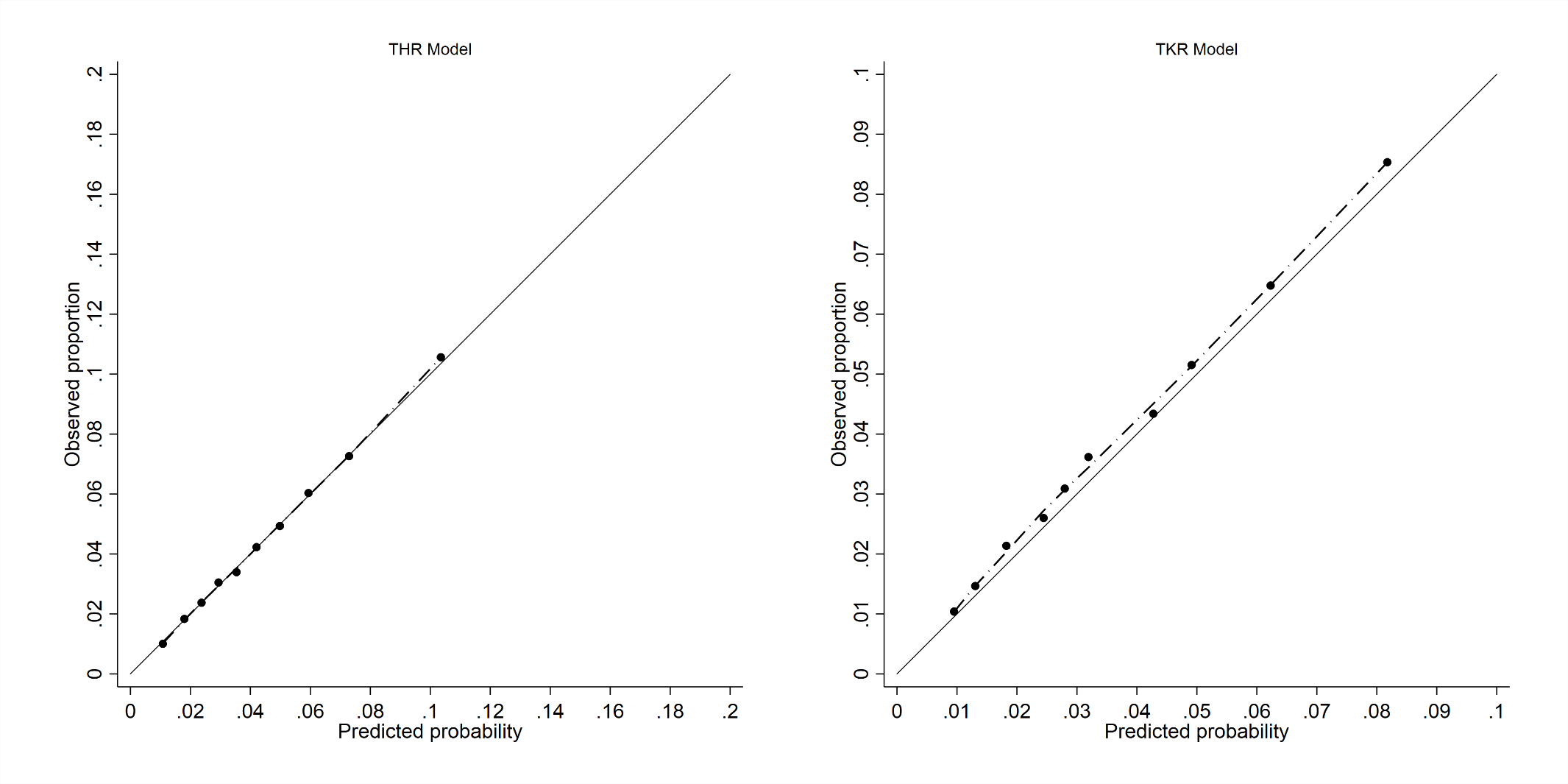


**Supplementary Table S1**. Source of predictors and the selection of predictors

| **QResearch** | **Systematic Review** | **Supplemented or emphasized by 1st round review** | **ReWAS study** | **Risk factor / potential predictor** | **Exclusion Votes** | **THR/TKR** | **Feasibility check and action notes** |
| --- | --- | --- | --- | --- | --- | --- | --- |
| ⚫ | ⚫ |  |  | Age | 1 | Both | included (inc) |
| ⚫ | ⚫ |  |  | Gender | 1 | Both | inc |
| ⚫ |  |  | ⚫ | Ethnicity | 1 | Both | inc |
|  |  | ⚫ |  | Region or trust | 1 | Both | inc |
| ⚫ |  | ⚫ |  | Deprivation (townsend score or SEIFA score) | 1 | Both | Not available (N/A) |
| ⚫ |  |  |  | Family history of outcome (knee or hip arthroplasty) | 2 | Both | N/A |
|  |  |  | ⚫ | Family history | 2 | Both | The 2 were combined to one indicator and restricted to the 2 read codes for FH Arthritis and FH Osteoarthritis |
|  |  |  | ⚫ | Family history yes | 3 | Both |  |
| ⚫ | ⚫ |  | ⚫ | Smoking Status | 0 | Both | inc |
| ⚫ |  |  | ⚫ | Drinking Status | 0 | Both | inc |
|  |  |  | ⚫ | Physical activity | 0 | Both | inc |
|  |  |  | ⚫ | Diet consultation | 2 | Both | inc |
|  |  |  | ⚫ | Health education consultation | 4 | Both | Drop |
|  |  | ⚫ |  | Charlson comorbidity index | 0 | Both | inc |
|  |  | ⚫ |  | Number of consultations | 0 | Both | inc |
|  |  | ⚫ |  | Number of referrals | 1 | Both | inc |
| ⚫ |  |  | ⚫ | Cardiovascular disease | 1 | Both | Use the subgroup of cardiovascular diseases as below rather than the general definition |
| ⚫ |  |  |  | Atrial fibrillation | 3 | Both | inc |
| ⚫ |  |  |  | Congestive cardiac failure | 2 | Both | inc |
| ⚫ |  |  |  | Valvular heart disease | 3 | Both | inc |
| ⚫ |  |  |  | Varicose veins | 4 | Both | drop |
| ⚫ |  |  |  | Venous thromboembolism | 3 | Both | inc |
| ⚫ |  |  | ⚫ | Treated hypertension | 3 | Both | inc |
|  |  |  | ⚫ | Diagnosis of respiratory diseases | 4 | Knee | Drop |
| ⚫ |  |  | ⚫ | Asthma | 3 | Knee | inc |
| ⚫ |  |  |  | COPD | 3 | Knee | inc |
| ⚫ |  |  |  | Chronic liver disease | 3 | Both | inc |
| ⚫ |  |  |  | Type 1 diabetes | 3 | Both | Combining type 1 diabetes and type 2 diabetes to diabetes mellitus |
| ⚫ |  |  |  | Type 2 diabetes | 2 | Both |  |
| ⚫ |  |  |  | Malabsorption | 3 | Both | inc |
| ⚫ |  |  |  | Inflammatory bowl diseases | 2 | Both | inc |
| ⚫ |  |  |  | Dementia | 3 | Both | inc |
|  |  | ⚫ |  | Other chronic neurological disease - e.g. multiple sclerosis, cerebral palsy, cerebrovascular disease | 3 | Both | Use multiple sclerosis, cerebral palsy and cerebrovascular disease as separate indictors |
|  |  | ⚫ | ⚫ | Mental problem: anxiety / depression | 3 | Both | inc |
| ⚫ |  |  | ⚫ | Rheumatoid arthritis | 0 | Knee | inc |
| ⚫ |  |  |  | Systemic lupus erythematosus | 2 | Both | inc |
| ⚫ |  |  |  | Falls | 0 | Both | inc |
|  |  |  | ⚫ | History of musculoskeletal disorders | 0 | Both | used the detailed definition as below |
| ⚫ |  | ⚫ |  | Previous Fracture/injury | 0 | Both | combining joint-specific fracture/injury and sprain as one indiactor |
|  | ⚫ | ⚫ | ⚫ | Previous Knee Injury | 0 | Both |  |
|  |  | ⚫ |  | Osteoporosis/osteopenia/osteoporotic fracture history | 1 | Both | inc |
|  |  | ⚫ |  | Referred (groin) pain | 1 | Both | N/A |
|  |  | ⚫ |  | Widespread pain / multiple consultations for different pain conditions | 1 | Both | N/A |
|  |  | ⚫ |  | Pain and positioning of pain in detecting hip osteoarthritis and its severity | 1 | Both | N/A |
|  |  | ⚫ |  | O/E- Knee effusion | 1 | Both | inc |
| ⚫ |  |  |  | Previous bleed | 3 | Both | inc |
|  |  |  |  | O/E - diabetic foot | 3 | Hip | inc |
|  | ⚫ |  | ⚫ | Height | 1 | Both | Using the BMI below as indictor rather than this two separate measurements |
|  | ⚫ |  | ⚫ | Weight | 0 | Both |  |
| ⚫ | ⚫ |  | ⚫ | BMI | 0 | Both | inc |
|  |  | ⚫ |  | Rheumatoid factor | 2 | Both | inc |
|  |  | ⚫ |  | Scoliosis / kyphosis | 1 | Both | inc |
|  |  | ⚫ |  | Developmental Dysplasia of the hip (DDH) | 0 | Both | inc |
|  |  | ⚫ |  | Chondrocalcinosis | 1 | Both | inc |
|  |  |  | ⚫ | Osteoarthritis and allied disorders | 0 | Both | inc, with joint specification |
|  |  |  | ⚫ | Arthropathy | 0 | Both | Drop due to the feasibility in the EHR |
|  |  |  | ⚫ | Non-specific osteoarthritis | 1 | Both | inc |
|  |  |  | ⚫ | Low back pain | 1 | Both | inc |
|  |  |  | ⚫ | Pain Consultation | 1 | Both | drop, the first joint pain consultation is the entry criteria |
|  |  | ⚫ |  | Polypharmacy | 1 | Both | inc |
| ⚫ |  | ⚫ | ⚫ | Knee joint injection | 0 | Knee | inc |
|  |  | ⚫ |  | Previous knee arthroscopy | 0 | Knee | inc |
|  |  | ⚫ |  | Previous ACL reconstruction | 0 | Knee | inc |
| ⚫ |  |  |  | Phenytoin | 3 | Both | inc |
|  |  |  | ⚫ | Physiotherapy or remedial therapy | 2 | Both | inc |
| ⚫ |  |  | ⚫ | Corticosteroids | 2 | knee | inc |
| ⚫ |  |  | ⚫ | Antidepressant drugs | 3 | Both | inc |
| ⚫ |  | ⚫ | ⚫ | Opioid Analgesics | 2 | Both | inc |
| ⚫ |  |  | ⚫ | Sex hormones | 3 | Both | inc |
|  |  |  | ⚫ | Prostaglandins & Oxytocics | 3 | Both | inc |
|  |  | ⚫ |  | Bishosphonates / Anti-osteoporosis drugs | 2 | Both | inc |
| ⚫ |  | ⚫ | ⚫ | NSAIDS or COX-2 | 2 | Both | inc |
|  |  |  | ⚫ | Topical NSAIDS or other topical creams | 2 | Both | inc |
|  |  |  | ⚫ | Glucocosteroids | 2 | Both | inc |

**Supplementary Table S2**. Multivariable adjusted hazard ratios (full model) and population attributable risk between each candidate predictor and total hip replacement in derivation cohort

|  | HR | 95% CI of HR | | EXPO | **PAR** | 95% CI of PAR | | Exclusion by PAR* |
| --- | --- | --- | --- | --- | --- | --- | --- | --- |
| Gender (male as reference) | 1.02 | 0.98 | 1.07 | 0.64 | 1.53% | -1.03% | 4.05% | 3 |
| Ethnicity (Whited as reference) |  |  |  |  |  |  |  |  |
| Not Recorded | 1.00 | 0.96 | 1.03 | 0.68 | -0.29% | -2.79% | 2.18% | 1 |
| Other ethnicity group | 0.37 | 0.28 | 0.49 | 0.01 | -0.76% | -0.87% | -0.62% | 1 |
| Family history of arthritis / osteoarthritis (no history as reference) | 1.11 | 0.91 | 1.35 | 0.01 | 0.07% | -0.06% | 0.21% | 2 |
| Smoking status (not-recorded/non-smoker/Ex-smoker as reference) |  |  |  |  |  |  |  |  |
| Light smoker | 0.65 | 0.55 | 0.76 | 0.02 | -0.81% | -1.04% | -0.54% | 1 |
| Moderate/Heavy smoker | 0.76 | 0.71 | 0.81 | 0.12 | -2.84% | -3.45% | -2.20% | 3 |
| Drinking status (not-recorded/non-drinker as reference) |  |  |  |  |  |  |  |  |
| Ex-drinker | 1.01 | 0.91 | 1.12 | 0.04 | 0.04% | -0.32% | 0.43% | 2 |
| Light drinker | 1.21 | 1.16 | 1.27 | 0.70 | 12.94% | 10.07% | 15.75% | 3 |
| Moderate drinker | 1.39 | 1.21 | 1.60 | 0.01 | 0.54% | 0.29% | 0.82% | 2 |
| Heavy drinker | 1.08 | 0.85 | 1.38 | 0.01 | 0.05% | -0.10% | 0.25% | 2 |
| Physical Activity (not-recorded/no physical activity as reference) |  |  |  |  |  |  |  |  |
| Taking light physical activity | 1.02 | 0.96 | 1.09 | 0.09 | 0.21% | -0.36% | 0.80% | 2 |
| Taking moderate physical activity | 1.09 | 1.01 | 1.17 | 0.06 | 0.53% | 0.08% | 1.00% | 2 |
| Taking heavy physical activity | 1.38 | 1.14 | 1.66 | 0.01 | 0.25% | 0.09% | 0.43% | 2 |
| Having diet consultation (no as reference) | 1.01 | 0.97 | 1.05 | 0.37 | 0.35% | -1.00% | 1.71% | 2 |
| Chronic Liver Disease (no as reference) | 0.99 | 0.88 | 1.12 | 0.02 | -0.01% | -0.27% | 0.28% | 1 |
| Diabetes Mellitus (no as reference) | 0.84 | 0.79 | 0.89 | 0.14 | -2.31% | -3.06% | -1.53% | 3 |
| Malabsorption (no as reference) | 0.82 | 0.62 | 1.08 | 0.00 | -0.09% | -0.19% | 0.04% | 1 |
| Inflammatory Bowel Disease (no as reference) | 0.95 | 0.88 | 1.03 | 0.06 | -0.27% | -0.70% | 0.18% | 1 |
| Dementia (no as reference) | 0.34 | 0.18 | 0.63 | 0.00 | -0.28% | -0.35% | -0.16% | 1 |
| Cerebral Palsy (no as reference) | 1.68 | 0.80 | 3.53 | 0.00 | 0.03% | -0.01% | 0.13% | 2 |
| Multiple Sclerosis (no as reference) | 0.75 | 0.51 | 1.11 | 0.00 | -0.08% | -0.15% | 0.04% | 1 |
| Cerebrovascular Disease (no as reference) | 0.85 | 0.76 | 0.95 | 0.04 | -0.60% | -0.94% | -0.22% | 1 |
| Mental Problem (no as reference) |  |  |  |  |  |  |  |  |
| Anxiety | 0.85 | 0.80 | 0.90 | 0.13 | -1.92% | -2.57% | -1.23% | 3 |
| Depression | 0.85 | 0.80 | 0.90 | 0.16 | -2.48% | -3.32% | -1.60% | 3 |
| Systemic Lupus erythematosus (no as reference) | 0.79 | 0.51 | 1.23 | 0.00 | -0.05% | -0.11% | 0.05% | 1 |
| Falls (no as reference) | 0.89 | 0.84 | 0.96 | 0.10 | -1.06% | -1.64% | -0.45% | 3 |
| Fracture, sprain, injury (no as reference) | 1.83 | 1.67 | 2.02 | 0.02 | 1.93% | 1.56% | 2.34% | 3 |
| Osteoporosis (no as reference) | 0.88 | 0.79 | 0.98 | 0.04 | -0.52% | -0.92% | -0.07% | 1 |
| Diabetic foot | 1.02 | 0.91 | 1.13 | 0.05 | 0.07% | -0.42% | 0.61% | 2 |
| Bleed (no as reference) | 0.95 | 0.90 | 1.00 | 0.15 | -0.71% | -1.44% | 0.05% | 1 |
| Scoliosis/kyphosis (no as reference) | 0.92 | 0.75 | 1.14 | 0.01 | -0.06% | -0.20% | 0.11% | 1 |
| Development Dysplasia of the Hip (no as reference) | 5.35 | 2.96 | 9.67 | 0.00 | 0.10% | 0.04% | 0.19% | 2 |
| Chondrocalcinosis (no as reference) | 0.90 | 0.64 | 1.27 | 0.00 | -0.03% | -0.09% | 0.07% | 1 |
| Osteoarthritis and allied disorders (no as reference) |  |  |  |  |  |  |  |  |
| Knee OA | 1.17 | 0.74 | 1.86 | 0.00 | 0.02% | -0.02% | 0.08% | 2 |
| Hand OA | 0.36 | 0.32 | 0.42 | 0.03 | -2.14% | -2.30% | -1.95% | 3 |
| Generalised OA | 0.54 | 0.47 | 0.61 | 0.03 | -1.21% | -1.38% | -1.01% | 3 |
| other joint OA | 0.43 | 0.39 | 0.48 | 0.04 | -2.39% | -2.59% | -2.17% | 3 |
| Non-specific OA (no as reference) | 0.48 | 0.46 | 0.50 | 0.41 | -27.20% | -28.55% | -25.89% | 3 |
| Low back pain (no as reference) | 0.99 | 0.96 | 1.03 | 0.50 | -0.39% | -2.22% | 1.45% | 1 |
| Hypertension (no as reference) | 1.00 | 0.96 | 1.04 | 0.34 | 0.01% | -1.30% | 1.34% | 2 |
| Artrial Fibriation (no as reference) | 1.00 | 0.90 | 1.11 | 0.03 | -0.01% | -0.34% | 0.35% | 1 |
| Congestive Cardiac Failure (no as reference) | 0.68 | 0.57 | 0.80 | 0.02 | -0.69% | -0.92% | -0.42% | 1 |
| Venous Thromboembolism (no as reference) | 1.02 | 0.91 | 1.13 | 0.03 | 0.05% | -0.23% | 0.35% | 2 |
| Valvular Heart Disease (no as reference) | 0.99 | 0.96 | 1.03 | 0.50 | -0.39% | -2.22% | 1.45% | 1 |
| Phenytoin (no as reference) | 0.95 | 0.66 | 1.37 | 0.00 | -0.01% | -0.09% | 0.10% | 1 |
| Physiotherapy (no as reference) | 1.07 | 1.01 | 1.14 | 0.09 | 0.65% | 0.10% | 1.23% | 2 |
| Glucocosteroids (no as reference) | 0.96 | 0.90 | 1.01 | 0.11 | -0.47% | -1.05% | 0.15% | 1 |
| Antidepressant (no as reference) | 0.96 | 0.92 | 1.01 | 0.36 | -1.30% | -2.95% | 0.37% | 3 |
| Analgesics (no as reference) |  |  |  |  |  |  |  |  |
| Weak combination opioids | 0.93 | 0.89 | 0.96 | 0.64 | -5.02% | -7.64% | -2.43% | 3 |
| Moderate combination opioids | 1.00 | 0.85 | 1.19 | 0.01 | 0.01% | -0.16% | 0.20% | 2 |
| Strong / very strong combination opioids | 1.07 | 0.88 | 1.30 | 0.01 | 0.07% | -0.12% | 0.30% | 2 |
| Hormone treatment (no as reference) | 0.95 | 0.91 | 1.00 | 0.27 | -1.25% | -2.44% | -0.03% | 3 |
| Bisphosphonates (no as reference) | 1.18 | 1.06 | 1.30 | 0.05 | 0.88% | 0.31% | 1.51% | 2 |
| Topical NSAIDS (no as reference) |  |  |  |  |  |  |  |  |
| NSAIDS | 0.73 | 0.70 | 0.77 | 0.23 | -6.41% | -7.28% | -5.51% | 3 |
| OTHER | 0.71 | 0.59 | 0.85 | 0.01 | -0.35% | -0.50% | -0.18% | 1 |
| Drugs for rheumatoid disease and gout (no as reference) |  |  |  |  |  |  |  |  |
| NSAIDS | 1.02 | 0.98 | 1.06 | 0.61 | 1.32% | -1.10% | 3.73% | 3 |
| COX2 | 1.06 | 0.99 | 1.14 | 0.07 | 0.41% | -0.07% | 0.92% | 2 |
| Prostaglandins & Oxytocics (no as reference) | 0.92 | 0.83 | 1.01 | 0.03 | -0.28% | -0.58% | 0.04% | 1 |
| Rheumatoid factor test (no as reference) | 1.03 | 0.76 | 1.37 | 0.00 | 0.01% | -0.07% | 0.11% | 2 |

***tag=1, PAR in (-1,0); tag=2, PAR in (0, 1); tag=3, PAR<=-1 or PAR>=1; predictors labelled as tag3 (or at least one level labelled as tag3 for categorical predictors) and not excluded by backward elimination remained in the final model.***

**Supplementary Table S3**. Multivariable adjusted hazard ratios (full model) and population attributable risk between each candidate predictor and total knee replacement in derivation cohort

|  | HR | 95% CI of HR | | EXPO | **PAR** | 95% CI of PAR | | Exclusion by PAR* |
| --- | --- | --- | --- | --- | --- | --- | --- | --- |
| Gender (male as reference) | 0.87 | 0.84 | 0.90 | 0.5734 | -8.23% | -10.44% | -6.02% | 3 |
| Ethnicity (Whited as reference) |  |  |  |  |  |  |  |  |
| Not Recorded | 1.03 | 0.99 | 1.06 | 0.6828 | 1.72% | -0.51% | 3.92% | 3 |
| Other ethnicity group | 0.88 | 0.76 | 1.03 | 0.0144 | -0.17% | -0.35% | 0.04% | 1 |
| Family history of arthritis / osteoarthritis (no history as reference) |  |  |  |  |  |  |  |  |
| FH of Arthritis/Osteoarthritis | 0.84 | 0.69 | 1.03 | 0.0051 | -0.08% | -0.16% | 0.01% | 1 |
| Smoking status (not-recorded/non-smoker/Ex-smoker as reference) |  |  |  |  |  |  |  |  |
| Light smoker | 0.77 | 0.65 | 0.91 | 0.0204 | -0.48% | -0.72% | -0.19% | 1 |
| Moderate/Heavy smoker | 0.75 | 0.71 | 0.80 | 0.1163 | -2.98% | -3.51% | -2.43% | 3 |
| Drinking status (not-recorded/non-drinker as reference) |  |  |  |  |  |  |  |  |
| Ex-drinker | 0.93 | 0.84 | 1.02 | 0.0335 | -0.25% | -0.53% | 0.05% | 1 |
| Light drinker | 1.12 | 1.07 | 1.16 | 0.7078 | 7.73% | 4.96% | 10.45% | 3 |
| Moderate drinker | 1.31 | 1.17 | 1.47 | 0.0157 | 0.49% | 0.26% | 0.74% | 2 |
| Heavy drinker | 1.01 | 0.84 | 1.21 | 0.0076 | 0.01% | -0.12% | 0.16% | 2 |
| Physical Activity (not-recorded/no physical activity as reference) |  |  |  |  |  |  |  |  |
| Taking light physical activity | 1.06 | 1.01 | 1.11 | 0.1079 | 0.66% | 0.14% | 1.19% | 2 |
| Taking moderate physical activity | 1.12 | 1.06 | 1.18 | 0.0713 | 0.82% | 0.41% | 1.26% | 2 |
| Taking heavy physical activity | 1.34 | 1.15 | 1.55 | 0.0078 | 0.26% | 0.12% | 0.43% | 2 |
| Having diet consultation (no as reference) | 1.01 | 0.97 | 1.04 | 0.1884 | 0.10% | -0.49% | 0.71% | 2 |
| Asthma (no as reference) | 1.08 | 1.01 | 1.14 | 0.1426 | 1.07% | 0.17% | 2.00% | 3 |
| COPD (no as reference) | 0.72 | 0.66 | 0.78 | 0.0483 | -1.38% | -1.65% | -1.09% | 3 |
| Chronic Liver Disease (no as reference) | 0.93 | 0.86 | 1.02 | 0.026 | -0.17% | -0.37% | 0.04% | 1 |
| Diabetes Mellitus (no as reference) | 0.79 | 0.74 | 0.84 | 0.1306 | -2.85% | -3.55% | -2.10% | 3 |
| Malabsorption (no as reference) | 0.86 | 0.70 | 1.06 | 0.005 | -0.07% | -0.15% | 0.03% | 1 |
| Inflammatory Bowel Disease (no as reference) | 0.92 | 0.87 | 0.97 | 0.0642 | -0.52% | -0.85% | -0.17% | 1 |
| Dementia (no as reference) | 0.78 | 0.62 | 0.98 | 0.0066 | -0.14% | -0.25% | -0.01% | 1 |
| Cerebral Palsy (no as reference) | 0.72 | 0.32 | 1.61 | 0.0005 | -0.01% | -0.03% | 0.03% | 1 |
| Multiple Sclerosis (no as reference) | 1.01 | 0.77 | 1.32 | 0.0031 | 0.00% | -0.07% | 0.10% | 2 |
| Cerebrovascular Disease (no as reference) | 0.89 | 0.82 | 0.98 | 0.0388 | -0.42% | -0.72% | -0.08% | 1 |
| Mental Problem (no as reference) |  |  |  |  |  |  |  |  |
| Anxiety | 0.78 | 0.74 | 0.82 | 0.135 | -3.09% | -3.63% | -2.53% | 3 |
| Depression | 0.85 | 0.82 | 0.90 | 0.0866 | -1.28% | -1.63% | -0.91% | 3 |
| Rheumatoid arthritis (no as reference) | 1.22 | 1.12 | 1.32 | 0.0245 | 0.53% | 0.30% | 0.78% | 2 |
| Systemic Lupus erythematosus (no as reference) | 0.89 | 0.64 | 1.25 | 0.0866 | -0.92% | -3.23% | 2.15% | 1 |
| Falls (no as reference) | 0.96 | 0.92 | 1.00 | 0.1198 | -0.50% | -1.01% | 0.03% | 1 |
| Fracture, sprain, injury (no as reference) |  |  |  |  |  |  |  |  |
| knee fracture/knee sprain/knee injury | 1.30 | 1.25 | 1.37 | 0.0568 | 1.70% | 1.38% | 2.04% | 3 |
| Osteoporosis (no as reference) | 0.95 | 0.87 | 1.03 | 0.0468 | -0.25% | -0.62% | 0.15% | 1 |
| Knee effusion (no as reference) | 1.11 | 1.02 | 1.20 | 0.015 | 0.16% | 0.03% | 0.30% | 2 |
| Bleed (no as reference) | 0.96 | 0.92 | 0.99 | 0.1696 | -0.76% | -1.39% | -0.11% | 1 |
| Scoliosis/kyphosis (no as reference) | 0.92 | 0.77 | 1.09 | 0.0079 | -0.07% | -0.18% | 0.07% | 1 |
| Development Dysplasia of the Hip (no as reference) | 0.62 | 0.16 | 2.49 | 0.0002 | -0.01% | -0.02% | 0.03% | 1 |
| Chondrocalcinosis (no as reference) | 1.24 | 1.05 | 1.46 | 0.0038 | 0.09% | 0.02% | 0.17% | 2 |
| Osteoarthritis and allied disorders (no as reference) |  |  |  |  |  |  |  |  |
| Hip OA | 0.59 | 0.55 | 0.63 | 0.064 | -2.72% | -2.99% | -2.44% | 3 |
| Hand OA | 0.61 | 0.56 | 0.68 | 0.0299 | -1.17% | -1.34% | -0.98% | 3 |
| Generalised OA | 0.74 | 0.68 | 0.81 | 0.0258 | -0.67% | -0.84% | -0.49% | 1 |
| other joint OA | 0.75 | 0.69 | 0.81 | 0.0364 | -0.92% | -1.13% | -0.69% | 1 |
| Non-specific OA (no as reference) | 1.17 | 1.12 | 1.22 | 0.3536 | 5.62% | 4.04% | 7.16% | 3 |
| Low back pain (no as reference) | 0.88 | 0.85 | 0.91 | 0.5257 | -6.81% | -8.59% | -5.03% | 3 |
| Hypertension (no as reference) | 0.95 | 0.92 | 0.99 | 0.3589 | -1.71% | -2.92% | -0.48% | 3 |
| Atrial Fibrillation (no as reference) | 0.86 | 0.79 | 0.93 | 0.0397 | -0.57% | -0.83% | -0.28% | 1 |
| Congestive Cardiac Failure (no as reference) | 0.77 | 0.68 | 0.88 | 0.0232 | -0.54% | -0.76% | -0.29% | 1 |
| Venous Thromboembolism (no as reference) | 1.06 | 0.99 | 1.14 | 0.0294 | 0.19% | -0.03% | 0.42% | 2 |
| Valvular Heart Disease (no as reference) | 0.88 | 0.78 | 1.00 | 0.0164 | -0.19% | -0.35% | -0.01% | 1 |
| Joint Injection (no as reference) | 1.65 | 1.59 | 1.72 | 0.102 | 6.24% | 5.68% | 6.81% | 3 |
| Knee Arthroscopy (no as reference) | 14.23 | 13.70 | 14.78 | 0.02 | 20.92% | 20.26% | 21.60% | 3 |
| ACL Reconstruction (no as reference) | 1.23 | 0.98 | 1.54 | 0.001 | 0.02% | 0.00% | 0.05% | 2 |
| Phenytoin (no as reference) | 0.90 | 0.68 | 1.18 | 0.003 | -0.03% | -0.10% | 0.05% | 1 |
| Physiotherapy (no as reference) | 1.00 | 0.96 | 1.05 | 0.0999 | 0.02% | -0.41% | 0.46% | 2 |
| Corticosteroids (no as reference) | 0.99 | 0.94 | 1.05 | 0.1493 | -0.09% | -0.93% | 0.78% | 1 |
| Glucocosteroids (no as reference) | 1.08 | 1.04 | 1.12 | 0.1241 | 0.98% | 0.48% | 1.51% | 2 |
| Antidepressant (no as reference) | 0.95 | 0.92 | 0.99 | 0.3843 | -1.91% | -3.35% | -0.46% | 3 |
| Analgesics (no as reference) |  |  |  |  |  |  |  |  |
| Weak combination opioids | 1.35 | 1.29 | 1.41 | 0.6452 | 18.44% | 15.74% | 21.09% | 3 |
| Moderate combination opioids | 1.39 | 1.23 | 1.56 | 0.0116 | 0.44% | 0.27% | 0.64% | 2 |
| Strong / very strong combination opioids | 1.64 | 1.49 | 1.80 | 0.0153 | 0.96% | 0.74% | 1.21% | 2 |
| Hormone treatment (no as reference) | 1.01 | 0.97 | 1.05 | 0.258 | 0.14% | -0.87% | 1.18% | 2 |
| Bisphosphonates (no as reference) | 1.03 | 0.96 | 1.11 | 0.058 | 0.18% | -0.26% | 0.64% | 2 |
| Topical NSAIDS (no as reference) |  |  |  |  |  |  |  |  |
| NSAIDS | 0.93 | 0.90 | 0.96 | 0.2709 | -1.90% | -2.82% | -0.96% | 3 |
| OTHER | 1.17 | 1.08 | 1.28 | 0.0178 | 0.31% | 0.14% | 0.49% | 2 |
| Drugs for rheumatoid disease and gout (no as reference) |  |  |  |  |  |  |  |  |
| NSAIDS | 1.40 | 1.34 | 1.47 | 0.6462 | 20.74% | 17.98% | 23.43% | 3 |
| COX2 | 1.26 | 1.17 | 1.35 | 0.0643 | 1.64% | 1.10% | 2.20% | 3 |
| Prostaglandins & Oxytocics (no as reference) | 0.93 | 0.87 | 1.00 | 0.0381 | -0.26% | -0.50% | -0.01% | 1 |
| Rheumatoid factor test (no as reference) | 0.76 | 0.58 | 1.00 | 0.0027 | -0.06% | -0.11% | 0.00% | 1 |

***tag=1, PAR in (-1,0); tag=2, PAR in (0, 1); tag=3, PAR<=-1 or PAR>=1; predictors labelled as tag3 (or at least one level labelled as tag3 for categorical predictors) and not excluded by backward elimination remained in the final model.***

**Supplementary Table S4. Sensitivity analysis: discrimination and calibration slope by applying risk algorithms in the sample incorporating patients with early (within 2 years since entry date) outcomes**

|  | C-statistics (95% CI) | Calibration slope (95% CI) |
| --- | --- | --- |
| THR cohort | 0.72 (0.72 to 0.73) | 0.99 (0.92 to 1.06) |
| TKR cohort | 0.77 (0.75 to 0.78) | 1.05 (1.02 to 1.08) |

**Supplementary Table S5. Sensitivity analysis: model coefficients by applying final predictors in the sample incorporating patients with early (within 2 years since entry date) outcomes**

| **Predictor** | **Subdistribution Hazard Ratio (95 CI)** | **Beta Coefficient** |
| --- | --- | --- |
| Final Model for primary total hip replacement | | |
| Gender: Women Vs Men | 1.00 (0.96, 1.04) | 0.002176 |
| Smoking status |  |  |
| Non-smoker/not recorded/Ex-smoker | reference |  |
| Light smoker | 0.64 (0.54, 0.76) | -0.44662 |
| Moderate / Heavy smoker | 0.75 (0.71, 0.80) | -0.28342 |
| Drinking status |  |  |
| Non-drinker/not recorded | reference |  |
| Ex-drinker | 1.01 (0.91, 1.11) | 0.008539 |
| Light drinker | 1.18 (1.14, 1.23) | 0.167596 |
| Moderate drinker | 1.36 (1.18, 1.55) | 0.304063 |
| Heavy drinker | 1.06 (0.85, 1.34) | 0.062208 |
| Diabetes Mellitus: yes vs no | 0.86 (0.81, 0.90) | -0.15431 |
| Mental disorders: yes vs no |  |  |
| No /Not recorded | reference |  |
| Anxiety | 0.85 (0.80, 0.90) | -0.16286 |
| Depression | 0.85 (0.81, 0.89) | -0.16449 |
| Falls | 0.85 (0.81, 0.91) | -0.15729 |
| Previous hip injury: yes vs no | 1.54 (1.41, 1.69) | 0.432443 |
| Recorded diagnosis of joint-specific osteoarthritis |  |  |
| No/not recorded | reference |  |
| Knee OA | 1.02 (0.65, 1.58) | 0.01519 |
| Hand OA | 0.21 (0.18, 0.23) | -1.58291 |
| Generalised OA | 0.29 (0.25, 0.33) | -1.24289 |
| Other joint OA | 0.23 (0.20, 0.26) | -1.47375 |
| Recorded diagnosis of non-specific Osteoarthritis: yes vs no | 0.27 (0.26, 0.28) | -1.31222 |
| Analgesics |  |  |
| No prescription | reference |  |
| Weak combination opioids | 0.93 (0.89, 0.97) | -0.07207 |
| Moderate combination opioids | 1.00 (0.86, 1.17) | 0.002591 |
| Strong / very strong combination opioids | 1.06 (0.88, 1.28) | 0.056439 |
| Antidepressant: yes vs no | 0.96 (0.92, 1.01) | -0.03609 |
| Topical NSAIDS |  |  |
| No prescription | reference |  |
| NSAIDS | 0.77 (0.73, 0.80) | -0.26674 |
| Other | 0.78 (0.66, 0.92) | -0.24756 |
| NSAIDS/COX2 |  |  |
| No prescription | reference |  |
| NSAIDS | 1.06 (1.02, 1.09) | 0.056441 |
| COX2 | 1.18 (1.11, 1.25) | 0.164300 |
| Hormone treatment: yes vs no | 0.96 (0.92, 1.00) | -0.03914 |
| (Age/10)^3 | - | 0.056923 |
| (Age/10)^3*ln(Age/10) | - | -0.02491 |
| (BMI/10)^2 | - | 0.137869 |
| (BMI/10)^3 | - | -0.02498 |
| ((Charlson Comorbidity Index+1)/10)^-2 | - | 0.00158 |
| ((Charlson Comorbidity Index+1)/10)^2 | - | -1.31862 |
| ((Number of referrals+1)/10)^-2 | - | -0.01552 |
| ((Number of referrals+1)/10)^-2*ln((Number of referrals+1)/10) | - | -0.00546 |
| ((Number of consultations+1)/1000)^-0.5 | - | -0.17164 |
| ((Number of consultations+1)/1000)^-0.5*ln((Number of consultations+1)/1000) | - | -0.0174 |
| ((Number of BNF chapters+1)/10)^-2 | - | 0.110529 |
| ((Number of BNF chapters+1)/10)^-2*ln((Number of BNF chapters+1)/10) | - | 0.046406 |
| Final Model for primary total knee replacement | | |
| Gender: Women Vs Men | 0.87 (0.85, 0.89) | -0.139496 |
| Ethnicity |  |  |
| White | reference | - |
| Other ethnicity group | 0.90 (0.88, 0.93) | -0.100227 |
| Not recorded | 1.04 (0.95, 1.12) | 0.034938 |
| Smoking status |  |  |
| Non-smoker/not recorded/Ex-smoker | reference | - |
| Light smoker | 0.75 (0.70, 0.80) | -0.292253 |
| Moderate / Heavy smoker | 0.75 (0.72, 0.77) | -0.294128 |
| Drinking status |  |  |
| Non-drinker/not recorded | reference | - |
| Ex-drinker | 1.13 (1.10, 1.17) | 0.122508 |
| Light drinker | 1.21 (1.110, 1.32) | 0.190747 |
| Moderate drinker/Heavy drinker | 1.34 (1.190, 1.51) | 0.293720 |
| Asthma, yes vs no | 1.07 (1.020, 1.12) | 0.064279 |
| COPD, yes vs no | 0.71 (0.680, 0.74) | -0.340306 |
| Diabetes mellitus: yes vs no | 0.88 (0.840, 0.92) | -0.128850 |
| Mental disorders: yes vs no |  |  |
| Anxiety | 0.76 (0.740, 0.78) | -0.270620 |
| Depression | 0.85 (0.830, 0.87) | -0.162216 |
| Previous knee injury: yes vs no | 1.29 (1.240, 1.34) | 0.254646 |
| Recorded diagnosis of joint-specific osteoarthritis |  |  |
| No/not recorded | reference | - |
| Hip OA | 0.59 (0.580, 0.61) | -0.521571 |
| Hand OA | 0.61 (0.590, 0.64) | -0.493812 |
| Generalised OA/ | 0.76 (0.730, 0.8) | -0.274384 |
| Other joint OA | 0.74 (0.710, 0.77) | -0.301054 |
| Recorded diagnosis of non-specific Osteoarthritis: yes vs no | 1.17 (1.150, 1.19) | 0.158083 |
| Low back pain: yes vs no | 0.87 (0.850, 0.89) | -0.138259 |
| Hypertension: yes vs no | 0.96 (0.940, 0.98) | -0.040532 |
| Joint Injection: yes vs no | 1.66 (1.590, 1.73) | 0.507833 |
| Knee Arthroscopy: yes vs no | 14.47 (10.580, 19.8) | 2.672140 |
| Antidepressant: yes vs no | 0.95 (0.930, 0.97) | -0.051037 |
| Analgesics |  |  |
| No prescription | reference | - |
| Weak combination opioids | 1.34 (1.30, 1.38) | 0.292199 |
| Moderate combination opioids | 1.37 (1.240, 1.52) | 0.317573 |
| Strong / very strong combination opioids | 1.59 (1.450, 1.75) | 0.464372 |
| Topical NSAIDS |  |  |
| No prescription | reference | - |
| NSAIDS | 0.94 (0.920, 0.96) | -0.065295 |
| Other | 1.18 (1.10, 1.26) | 0.162558 |
| NSAIDS/COX2 |  |  |
| No prescription | reference | - |
| NSAIDS | 1.42 (1.370, 1.47) | 0.350504 |
| COX2 | 1.27 (1.210, 1.34) | 0.240079 |
| (Age/10)^3 | - | 0.029616 |
| (Age/10)^3*ln(Age/10) | - | -0.132653 |
| (BMI/10)^2 | - | 0.281292 |
| (BMI/10)^3 | - | -0.047230 |
| ((Charlson Comorbidity Index+1)/10)^-2 | - | 0.002391 |
| ((Charlson Comorbidity Index+1)/10)^2 | - | -1.440470 |
| ((Number of referrals+1)/10)^-2 | - | -0.010520 |
| ((Number of referrals+1)/10)^-2*ln((Number of referrals+1)/10) | - | -0.002960 |
| ((Number of consultations+1)/1000)^-0.5 | - | -0.541070 |
| ((Number of consultations+1)/1000)^-0.5*ln((Number of consultations+1)/1000) | - | -0.073452 |

**Supplementary Table S6. Clinical examples for total hip replacement model**

| **Predictor** | **Clinical Examples** | |
| --- | --- | --- |
|  | **Patient A** | **Patient B** |
| CIF_0_ (t=10 years) | 6.8% | 6.8% |
| Age, year | 70 | 60 |
| Gender | Male | Female |
| Smoking status | Non-smoker | Non-smoker |
| Drinking status | Light drinker | Light drinker |
| Diabetes Mellitus | No | N0 |
| Mental disorders | No | No |
| Falls | No | No |
| Previous hip injury | Yes | No |
| Recorded diagnosis of joint-specific osteoarthritis | No | No |
| Recorded diagnosis of non-specific osteoarthritis | No | No |
| Analgesics | No | Weak combination opioids |
| Topical NSAIDS | No | No |
| NSAIDS/COX2 | NSAIDS | NSAIDS |
| Hormone treatment | No | Yes |
| Body mass index, kg/m2 | 24.8 | 35.1 |
| Charlson Comorbidity Index | 2 | 1 |
| Number of referrals | 2 | 0 |
| Number of consultations | 20 | 35 |
| Number of BNF chapters | 1 | 7 |
| **10-year risk of THR** | 79.7% | 62.4% |
|  | High risk | High risk |

**Supplementary Table S7. Clinical examples for total knee replacement model**

| **Predictor** | **Clinical Examples** | |
| --- | --- | --- |
|  | **Patient A** | **Patient B** |
| CIF_0_ (t=10 years) | 7.5% | 7.5% |
| Age, years | 58 | 70 |
| Gender | Male | Female |
| Ethnicity | Not Recorded | White |
| Smoking status | Non-smoker | Non-smoker |
| Drinking status | Non-drinker | Non-drinker |
| Asthma | No | No |
| COPD | No | No |
| Diabetes Mellitus | No | No |
| Mental disorders | No | No |
| Previous knee injury | No | No |
| Recorded diagnosis of joint-specific osteoarthritis | No | No |
| Recorded diagnosis of non-specific osteoarthritis | Yes | No |
| Low back pain | Yes | No |
| Hypertension | No | No |
| Joint Injection | No | No |
| Knee Arthroscopy | Yes | No |
| Antidepressant | No | No |
| Analgesics | No | Weak combination opioids |
| Topical NSAIDS | No | No |
| NSAIDS/COX2 | No | NSAIDS |
| Body mass index, kg/m2 | 26.0 | 42.0 |
| Charlson Comorbidity Index | 0 | 0 |
| Number of referrals | 2 | 1 |
| Number of consultations | 26 | 30 |
| **10-year risk of TKR** | 87.0% | 31.7% |
|  | High risk | Low risk |
